# Identification of functions linking quorum sensing with biofilm formation in *Burkholderia cenocepacia* H111

**DOI:** 10.1002/mbo3.24

**Published:** 2012-06

**Authors:** Silja Inhülsen, Claudio Aguilar, Nadine Schmid, Angela Suppiger, Kathrin Riedel, Leo Eberl

**Affiliations:** 1 Department of Microbiology, Institute of Plant Biology, University of ZurichZollikerstrasse, 107, 8008 Zurich, Switzerland; 2 Institute of Microbiology, Ernst-Moritz-Arndt University of GreifswaldFriedrich-Ludwig-Jahn-Strasse 15, D-17487, Greifswald,, Germany

**Keywords:** Biofilm, *Burkholderia cenocepacia*, microarray, proteomics, quorum sensing

## Abstract

*Burkholderia cenocepacia* has emerged as an important pathogen for patients suffering from cystic fibrosis (CF). Previous work has shown that this organism employs the CepIR quorum-sensing (QS) system to control the expression of virulence factors as well as the formation of biofilms. To date, however, very little is known about the QS-regulated virulence factors and virtually nothing about the factors that link QS and biofilm formation. Here, we have employed a combined transcriptomic and proteomic approach to precisely define the QS regulon in our model strain *B. cenocepacia* H111, a CF isolate. Among the identified CepR-activated loci, three were analyzed in better detail for their roles in biofilm development: (i) a gene cluster coding for the BclACB lectins, (ii) the large surface protein BapA, and (iii) a type I pilus. The analysis of defined mutants revealed that BapA plays a major role in biofilm formation on abiotic surfaces while inactivation of the type I pilus showed little effect both in a static microtitre dish-based biofilm assay and in flow-through cells. Inactivation of the *bclACB* lectin genes resulted in biofilms containing hollow microcolonies, suggesting that the lectins are important for biofilm structural development.

## Introduction

*Burkholderia cenocepacia* is one of the currently 17 validly described species that comprise the *Burkholderia cepacia* complex (Bcc) (Vanlaere et al. 2008). Bcc strains inhabit a wide variety of environmental niches and have been isolated from soil, water, rhizospheres, industrial settings, cosmetics, and presumed sterile solutions (Coenye and Vandamme 2003; Mahenthiralingam et al. 2005, 2008). Members of the Bcc complex have enormous biotechnological potential and were previously used to protect commercially important crops against fungal diseases. However, the use of Bcc strains in commercial applications has been severely limited by the U.S. Environmental Protection Agency, as Bcc species have also emerged as opportunistic pathogens of humans, particularly those affected by cystic fibrosis (CF) (Parke and Gurian-Sherman 2001; Vandamme et al. 2007; Mahenthiralingam et al. 2008). All Bcc species have been isolated from the environment as well as from CF sputa and at present no prediction of the pathogenic potential of a strain can be made solely on the basis of its phylogenetic status (Baldwin et al. 2007; Mahenthiralingam et al. 2008).

Like many Gram-negative bacteria, Bcc strains employ *N*-acyl-homoserine lactone (AHL)-dependent quorum sensing (QS) systems to express certain functions only when a critical population density has been attained. All investigated Bcc strains contain the CepIR system and additional QS systems have been identified in some strains (for reviews see Eberl 2006; Sokol et al. 2007). CepI has been shown to be responsible for the production of *N*-octanoyl homoserine lactone (C_8_-HSL) and minor amounts of *N*-hexanoyl homoserine lactone (C_6_-HSL). On reaching a particular threshold concentration, the AHLs bind to their cognate LuxR-type receptor protein CepR, which, in turn, leads to the induction or repression of target genes. Previous work has identified several QS-regulated functions in Bcc strains, including swarming motility, the production of extracellular (EC) proteases, chitinases, a polygalacturonase, and the biosynthesis of siderophores (Eberl 2006; Sokol et al. 2007). *Burkholderia cenocepacia* H111 mutants defective in QS were also shown to form only a flat and undifferentiated biofilm on abiotic surfaces when compared to the one of the wild type (WT) (Huber et al. 2001). A detailed quantitative analysis of the biofilm architectures revealed that the CepIR system is not involved in the regulation of initial cell attachment but rather controls the late stages of biofilm development (Huber et al. 2001, 2002). Employing a quorum quenching approach, that is the enzymatic degradation of AHL signal molecules, it was shown that QS regulates biofilm formation not only in *B. cenocepacia* but also in the large majority of strains investigated, belonging to nine Bcc species (Wopperer et al. 2006). However, the QS-regulated factors that would directly link QS and biofilm formation are unknown. In this study, we have employed a combined transcriptomic and proteomic approach to precisely map the QS regulon of *B. cenocepacia* H111. Three of the identified QS-regulated functions, a type I pilus (BCAL1677–BCAL1681), the BclACB lectins (BCAM0184–BCAM0186), and the large surface protein BapA (BCAM2140–BCAM2143), potentially linked QS with biofilm formation. The analysis of defined mutants in these functions revealed that BapA is of particular importance for biofilm formation on abiotic surfaces while little, if any, effect was observed when the *fimA* gene encoding the type I pilus structural gene (BCAL1677) was inactivated. The biofilm formed by a mutant in which all three AHL-regulated lectins were deleted exhibited a very characteristic biofilm structure with internal spherical regions that remained uncolonized.

## Results

### Mapping the CepR regulon of *B. cenocepacia* H111 by transcriptomics

**Figure 1:**
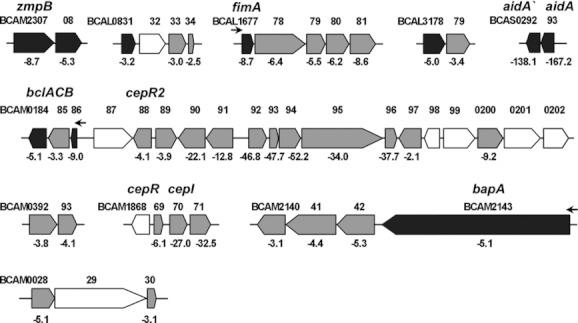
Genetic organization of selected QS-regulated genes in *B. cenocepacia* H111. Genes are depicted as arrows pointing in the direction of transcription. Fold change values are indicated below individual genes and are based on the comparison of the transcriptome of the *cepR* mutant H111-R with the one of the WT strain. Genes that were positively regulated by CepR in the transcriptome analysis are shown in gray, genes that were also identified as QS-regulated in the iTRAQ study are depicted in black, and genes with no change in expression are presented as open arrows. Numbers refer to the annotated genes of *B. cenocepacia* J2315 (Holden et al. 2009). The presence of CepR binding sites has been demonstrated/reported for the promoter regions of BCAL0831 2006, BCAM1868 2006, BCAM0189 (Malott et al. 2009), and BCAS0293 2004. Small black arrows represent the regions and orientation of the fragments used to generate promoter fusions of BCAL1677, BCAM0186, and BCAM2143.

To identify CepR-regulated genes in *B. cenocepacia* H111, we compared the transcriptome of the WT strain with the one of the *cepR* mutant H111-R using a custom *B. cenocepacia* oligonucleotide microarray. As a control, we included the complemented *cepR* mutant H111-R (pBAH27) in these experiments. Total RNA was extracted when the cultures reached a “quorate” state, which was defined as the time point at which transcription of *aidA*, a stringently CepR-regulated gene encoding a protein required for nematode pathogenicity, was maximal (OD_600_ of 2.5) (Fig. S1). We found 48 genes exhibiting a ≥threefold reduction in transcript levels in the *cepR* mutant and these are listed in Table 1. In addition, nine genes were ≥threefold upregulated in the mutant background (Table S4). Several of the CepR-activated genes appear to be organized in operons, as they are transcribed in the same orientation and show similar levels of regulation (Fig. 1). Complementation of H111-R with *cepR* on plasmid pBAH27 rescued expression of genes in the mutant strain in most cases (Table 1). Notably, several of the CepR-activated genes were found to be even more strongly expressed in the complemented *cepR* mutant than in the WT, probably due to the increased copy number of *cepR*. Some of these upregulated genes were only slightly downregulated in the *cepR* mutant H111-R (Table 1), suggesting that expression of these genes is low or that it may be higher at another time point during growth. Alternatively, activation of these genes may be the result of unspecific binding of CepR to operator sequences.

**Table 1:** Classification of a selection of QS-regulated genes and proteins by functional groups

		Transcriptome* (fold changes)		Proteome††
			
Class	Gene name or ID§	H111-R versus WT	H111-R (*cepR*^+^) versus WT	H111-R and H111-I
Cell attachment,motility, and membrane components	**BCAL1677 (*fimA*)**	−8.7	n.d.	↓
	BCAL1678	−6.4	n.d.	n.d.
	BCAL1679	−5.5	n.d.	n.d.
	BCAL1680	−6.2	n.d.	n.d.
	**BCAM0184 (*bclB*)**	−5.1¶	3.3	↓
	BCAM0185 (*bclC*)	−3.3¶	n.d.	n.d.
	**BCAM0186** (*bclA*)	−9.0	5.1¶	↓‡
	BCAM2140	−3.1¶	2.4	n.d.
	BCAM2141	−4.4¶	n.d.	n.d.
	BCAM2142	−5.3¶	n.d.	n.d.
	BCAM2143 (*bapA*)	−5.1¶	2.6	↓
Energy production and conversion	BCAL3179 (*ldhA*)	−3.4¶	2.6	n.d.
	BCAL3285 (*hmpA*)	−25.8	n.d.	n.d.
Defense mechanisms	BCAM0200	−9.2¶	15.0¶	n.d.
	BCAM0393	−4.1¶	5.6¶	n.d.
	**BCAS0293 (*aidA*)**	−167.2¶	24.0¶	↓
	**BCAS0292 (*aidA*’)**	−138.1¶	7.8¶	↓
Cell envelope biogenesis	BCAL1813	−5.3¶	4.1¶	n.d.
	BCAM2720	−4.4	−2.3	n.d.
	BCAS0498	−4.9	−2.4	n.d.
Signal transduction and secondary metabolites	BCAM0028	−5.1	n.d.	n.d.
	**BCAM1870** (*cepI*)	−27.0¶	n.d.	n.d.
Transport and metabolism	BCAL0121 (*aqpZ*)	−3.3¶	n.d.	n.d.
	BCAL0358	−4.7	2.5	↓
	**BCAL0833 (*phbB*)**	−3.0	4.6	n.d.
	BCAM0190	−22.1¶	6.0	n.d.
	**BCAM0191**	−12.8¶	3.5¶	n.d.
	BCAM0195	−34.0¶	6.9¶	n.d.
	BCAM0392	−3.8¶	3.9¶	n.d.
	**BCAM2307 (*zmpB*)**	−8.7¶	6.8	↓
	BCAM2308	−5.3¶	5.7	↓‡
	**BCAS0409 (*zmpA*)**	−4.4	3.5	↓
Transcriptional regulators	BCAL3178	−5.0¶	n.d.	n.d.
	BCAM0188 (*cepR2*)	−4.1¶	4.5¶	n.d.
	**BCAM0189**	−3.9	2.4	n.d.
	BCAM0835	−4.3	3.5	n.d.
Hypothetical proteins and others	BCAL0510	−5.7	n.d.	n.d.
	**BCAL0831**	−3.2	2.3	↓
	BCAL1681	−8.6	n.d.	n.d.
	BCAL1921	−3.5	n.d.	n.d.
	BCAM0030	−3.1	4.2¶	n.d.
	BCAM0192	−46.8¶	6.2	n.d.
	BCAM0193	−47.7¶	6.8	n.d.
	BCAM0194	−52.2¶	8.4	n.d.
	BCAM0196	−37.7¶	6.3¶	n.d.
	**BCAM1869**	−6.1¶	n.d.	n.d.
	**BCAM1871**	−32.5¶	2.3	n.d.
	BCAS0236	−5.4	−2.1	n.d.

* Genes whose expression is at least threefold reduced in the *B. cenocepacia* H111-R (*cepR* mutant strain) transcriptome relative to the wild type.

† Decrease in extracellular (EC) or whole cell (WC) protein amounts in the H111-R and H111-I (*cepI* mutant strain) proteome relative to the wild type (indicated by an arrow).

‡ Downregulated only in the proteome of H111-R (see Table S1).

¶ Significantly regulated genes (*P* < 0.05) as determined by ANOVA and the “Benjamini and Hochberg False Discovery Rate (BH_FDR) multiple testing correction”; n.d., not detected.

§ Gene names or ID refer to the annotated genes of *B. cenocepacia* J2315 (Holden et al. 2009).

*B. cenocepacia* K56-2 QS-regulated genes which were identified in a previous study are displayed in bold type (O'Grady et al. 2009).

### Identification of QS-regulated proteins in *B. cenocepacia* H111

To confirm and extend our transcriptomic analysis, we also compared the proteome of the WT with the one of mutant H111-R and the proteomes of the *cepI* mutant H111-I grown in the presence or absence of 200 nM C_8_-HSL. EC and whole cell (WC) protein fractions were extracted and analyzed by a gel-free quantitative proteomics approach (Isobaric Tag for Relative and Absolute Quantitation [iTRAQ] analysis). Only proteins identified in two independent proteome analyses (a and b, see Experimental Procedures), which were found to be at least twofold differentially expressed when comparing WT and mutant strains were considered as QS regulated (Table S1). Using iTRAQ coupled with tandem mass spectrometry (MS/MS), we were able to identify 1257 proteins in total, of which 60 were downregulated in the *cepR* mutant. From this set of 60 proteins, we detected 22 proteins that were also downregulated in the *cepI* mutant. When the *cepI* mutant was grown in medium supplemented with 200 nM, C_8_-HSL protein expression was in many cases restored to the levels of the WT.

We could detect 11 genes encoding differentially expressed proteins that were also among the ≥threefold positively QS-regulated genes identified in the transcriptome analysis (Table 1; Table S1). The most stringently regulated factors were identified in both the transcriptome and the proteome analysis and these include many genes that have also been identified as QS-regulated by transcriptomics in *B. cenocepacia* K56-2 (O'Grady et al. 2007) or by alternative methods in various Bcc strains (Table 1) (for a review, see Sokol et al. 2007). Interestingly, expression of some of the identified genes in our study has not previously been reported as QS-regulated, suggesting strain-specific differences of the CepIR regulons. The following genes with a ≥threefold decrease in expression levels were found to be QS-regulated only in strain H111: BCAL0121, BCAL0510, BCAL1678–BCAL1681, BCAL1813, BCAL1921, BCAL3178, BCAL3179, BCAL3285, BCAM0028, BCAM0030, BCAM0393, BCAM0835, BCAM2140–BCAM2143, BCAM2308, BCAM2720, BCAS0236, and BCAS0498 (Table 1).

We found that a large proportion of the QS-regulated proteins was not among the QS-regulated genes identified by the microarray analysis, suggesting that expression of many of the identified proteins may be controlled by QS at the posttranscriptional level. Similar observations have been made previously in the case of QS-regulated proteins in *Pseudomonas aeruginosa* PAO1 (Arevalo-Ferro et al. 2003).

As previous work has demonstrated that biofilm formation of *B. cenocepacia* H111 is CepIR-dependent (Huber et al. 2001), we were interested in identifying factors that would potentially link QS with the formation of biofilms, focusing on proteins believed to be EC. In this respect, three loci were of particular interest, as they encode functions previously implicated in the formation of biofilms: (i) a cluster of three genes encoding lectins (*bclACB*; BCAM0184–BCAM0186), (ii) a gene cluster encoding the large surface protein BapA (BCAM2143) and a type I secretion system (BCAM2140–BCAM2142), and (iii) a putative operon encoding a type I pilus structural gene and a chaperone/usher secretion apparatus (BCAL1677–BCAL1681) (Fig. 1). These three loci were chosen for more detailed analyses.

**Figure 2:**
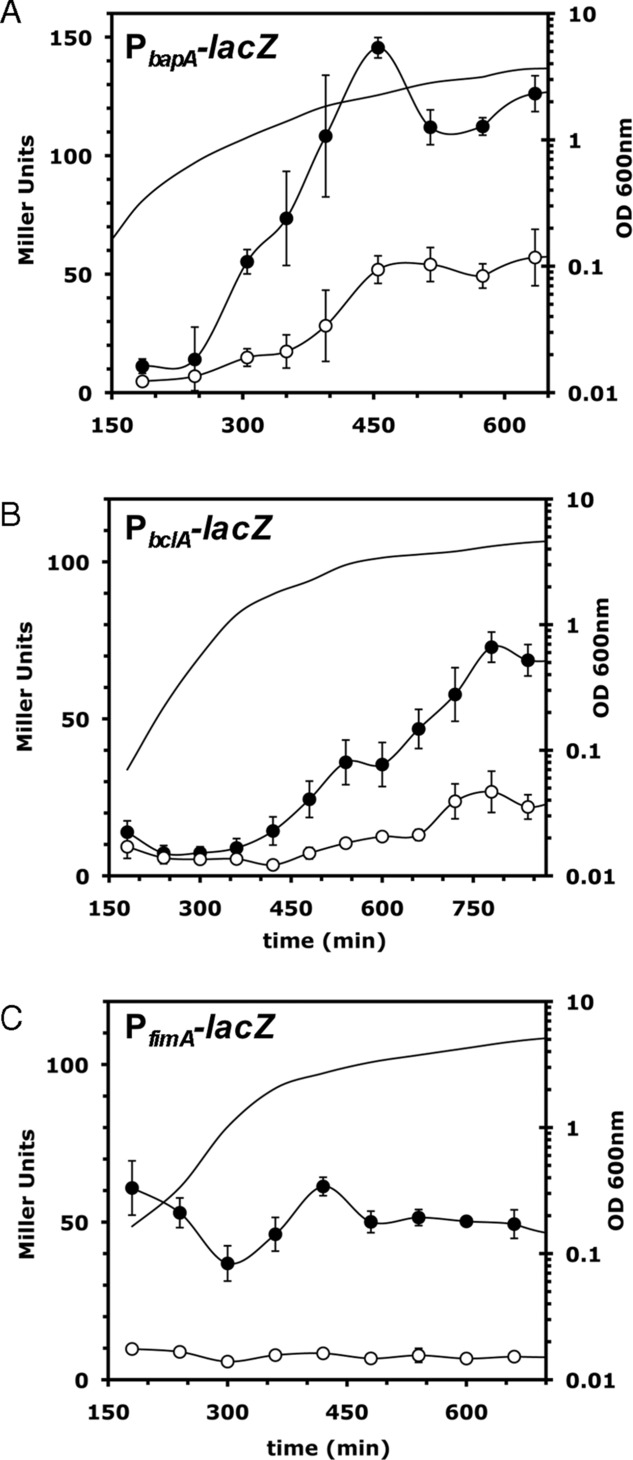
Transcription of *bclA, bapA* and *fimA* is positively regulated by CepR. ß-galactosidase activities of transcriptional fusions of the *bclA* (A), *bapA* (B), and *fimA* (C) promoter regions to *lacZ* were assayed in the *B. cenocepacia* H111 wild type (filled symbols) and the isogenic *cepR* mutant H111-R (open symbols) throughout the growth curve (solid line). Strains harboring the P*_bclA_-lacZ* and P*_bapA_-lacZ* fusions were grown in unbuffered LB medium, whereas strains used to measure the activity of the P*_fimA_-lacZ* fusion were grown in LB medium adjusted to pH 5 and 17 mM NaCl. Values are means ± SEM, *n* = 3.

### The large surface protein BapA plays a major role in biofilm formation

Our combined transcriptome and proteome analysis revealed that expression of BCAM2143 (*bapA* or *adhA*) is QS-regulated. This gene encodes a protein that belongs to a family of large surface proteins, many of which have been shown to be involved in biofilm formation in various bacterial species (Latasa et al. 2005). In fact, BCAM2143 was identified in a previous screen for transposon insertion mutants of *B. cenocepacia* H111 that were defective in biofilm formation. This gene was shown to play an important role in biofilm development and it was therefore named *bapA* (biofilm-associated protein) (Huber et al. 2002). Members of this protein family are normally transported via a type I secretion system to the cell surface (Hinsa et al. 2003). In fact, a cluster of three genes (BCAM2140–BCAM2142) encoding an ATP-binding cassette (ABC) transporter (type I secretion) is located downstream of *bapA* (Holden et al. 2009). Intriguingly, these three genes were also found to be QS-activated (Table 1; Fig. 1), and RT-PCR (Where PCR is polymerase chain reaction) performed over cDNA indicates that *bapA* and the downstream ABC transporter are part of a single transcriptional unit (Fig. S2). A P*_bapA_-lacZ* promoter fusion was constructed and assayed in the WT and in the *cepR* mutant background (Fig. 2). In full agreement with the microarray and proteome data, β-galactosidase activity was decreased to about one-third of the WT level in the absence of *cepR*.

**Figure 3:**
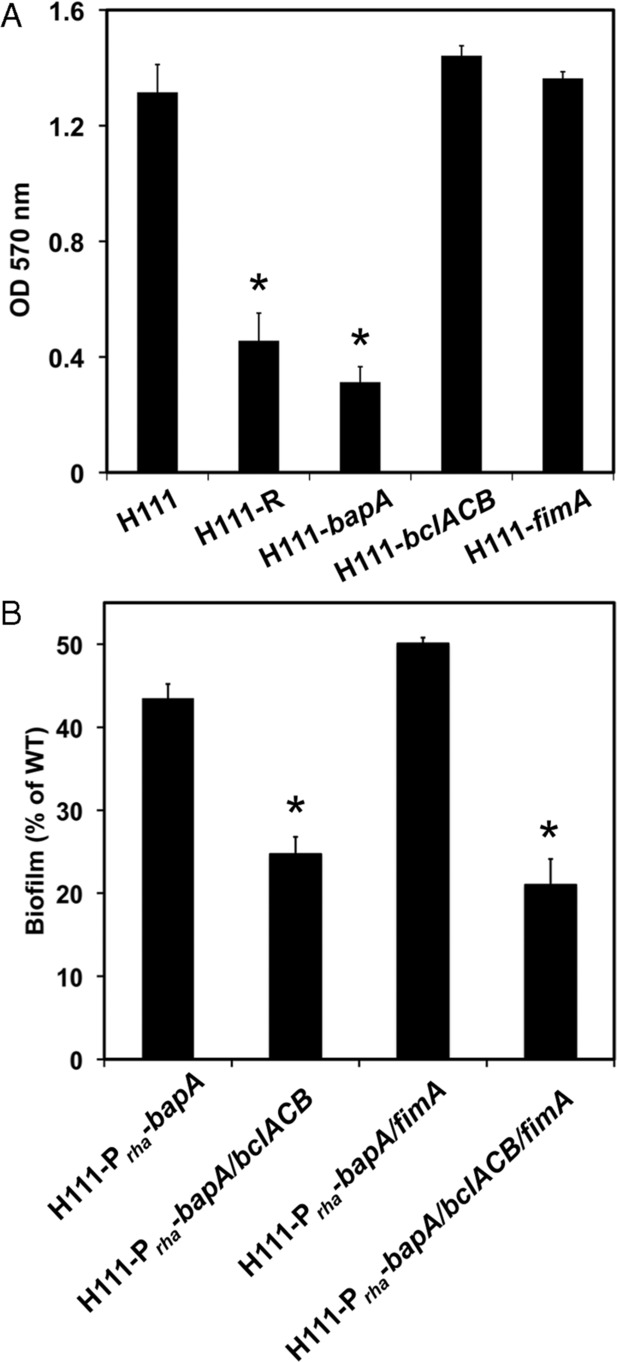
BapA has a strong influence on biofilm formation. (A) Biofilm formation in AB minimal media supplemented with 10 mM sodium citrate. After incubation at 30°C for 48 h, planktonic cells were removed and attached cells were stained with crystal violet as described in Methods section; defective biofilms were only observed with the *cepR* mutant H111-R and the *bapA* mutant H111-*bapA*. (B) BapA masks the effect of *bclACB*; *bapA* was expressed from a rhamnose-inducible promoter in strain H111-P*_rha_-bapA*. Values were adjusted to percentage of the wild type (WT). Asterisk indicates statistical significance (*t*-test, *P* < 0.01). Error bars indicate SEM, *n* = 3.

Mutant H111-*bapA*, in which *bapA* has been deleted, exhibited a diminished capacity to form a biofilm on the plastic surface of a microtitre dish (Fig. 3). Likewise, inactivation of the *bapA*-adjacent genes BCAM2140 or BCAM2141 reduced the ability to form biofilms to the level of H111-*bapA* (Fig. S3). These data strongly suggest that the gene cluster BCAM2140–BCAM2142 encodes an ABC transporter that is required for BapA export. To obtain a more complete picture of the role of BapA in biofilm formation, we engineered a strain of *B. cenocepacia* in which the expression of *bapA* was under the control of an inducible promoter. This was necessary since complementing with the complete operon (∼14 kbp) from a plasmid was not feasible. For this reason, the native promoter of *bapA* was exchanged with the rhamnose-inducible P*_rha_* promoter as detailed in the Material and Methods. The expression of the *bapA* operon in this conditional mutant was strictly dependent on the addition of rhamnose. This strain showed an increase in biofilm production when incremental amounts of rhamnose were added to the medium (Fig. S4).

We also analyzed biofilm formation of the *bapA* mutant in flow-through cells, which allows following biofilm development and determining biofilm structures (Christensen et al. 1999). In full agreement with the previous analysis of two *bapA* transposon insertion mutants (Huber et al. 2002), we observed that the microcolonies formed after 48 h of incubation are smaller and less abundant when compared to the biofilm of the WT strain (Fig. S5). This observation was confirmed and quantitated by employing the COMSTAT software package (http://www.comstat.dk; Heydorn et al. 2000; Fig. S5). In addition, a 72-h-old WT biofilm covered the entire glass surface whereas the *bapA* mutant grew in well-separated cell aggregates with only very few cells colonizing the interspace regions giving rise to a porous and disconnected biofilm (Fig. S5).

### BapA is associated with the bacterial cell surface

**Figure 4:**
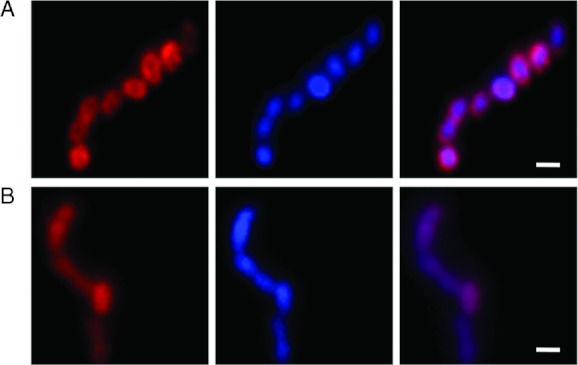
BapA is associated to the bacterial cell wall. *B. cenocepacia* wild-type strain H111 (A) and the *bcam2141* transporter mutant (B) were engineered to express a mCherry-BapA fusion protein when rhamnose is present in the culture medium. Samples were grown in M9 minimal media with rhamnose as sole carbon source for maximal expression of the fusion protein. Fluorescence emission was analyzed for mCherry (left), DAPI (middle), and merged (right). Bars, 1 μm.

The finding that BapA has a strong influence on biofilm development in *B. cenocepacia* H111 suggests that this protein has an important role in the biofilm matrix and that it may be localized on the bacterial cell surface. To test this hypo-thesis, we engineered a recombinant strain that expresses a mCherry-BapA fusion protein when the culture medium is supplemented with rhamnose (see Experimental Procedures for details). This alternative method was developed since we have been unable to raise functional antibodies against BapA. The mCherry-BapA protein fusion was transferred to the genetic backgrounds of both the WT strain and the *bcam2141* transporter mutant. As depicted in Figure 4, microscopic inspection revealed that the mCherry-BapA fusion protein gave rise to a fluorescent halo around the cell in the WT but not the *bcam2141* mutant background. Further evidence for a surface localization of BapA was obtained in a shotgun proteomics analysis of purified membrane proteins (data not shown).

### Expression of the BclACB lectins is controlled QS

Expression of the three *bclACB* genes was 9.0-, 3.3-, and 5.1-fold, respectively, downregulated in the *cepR* mutant H111-R relative to the WT (Table 1; Fig. 1). BclA and BclB were also identified as positively QS-regulated proteins (Table 1; Table S1). These data suggest that the three genes form an operon in *B. cenocepacia* H111. To test this hypothesis, we performed RT-PCR using cDNA as template. The results indicate that these genes are indeed organized as an operon (Fig. S2). To further analyze the expression of the operon, we fused the promoterless *lacZ* gene to the *bclA* upstream region (first gene in the operon) and measured β-galactosidase activities along the growth curve. In the WT background, the promoter activity was low during exponential growth but increased at the transition to the stationary phase (Fig. 2). Induction of the P*_bclA_-lacZ* fusion was found to be approximately twofold reduced in stationary phase in strain H111-R, confirming that the activity of the promoter is CepR-dependent.

We also studied the expression of the lectins using polyclonal antibodies raised against purified BclB protein, the lectin encoded by the last gene of the *bclACB* operon. Liquid cultures of the WT H111, the lectin-deficient mutant H111-*bclACB*, the *cepR* mutant H111-R, and the complemented *cepR* mutant H111-R (*cepR*^+^) were analyzed by Western blot. Expectedly, expression of BclB was found to be abolished in both H111-*bclACB* and H111-R. Importantly, expression of BclB in the QS-deficient mutant H111-R could be completely restored when the strain was complemented with the WT *cepR* allele on a plasmid. We also observed that, in these conditions, BclB expression was transient and was only observed at the onset of the stationary phase but not in overnight cultures (Fig. S6).

### The BclACB lectins are required for biofilm structural development

Previous work in *P. aeruginosa* has shown that the PA-IIL lectin, whose expression is regulated by QS and the RNA polymerase sigma factor RpoS, is important for biofilm formation (Winzer et al. 2000; Tielker et al. 2005; Johansson et al. 2008). We next tested whether the *B. cenocepacia* H111 lectins are similarly required for biofilm formation. Using a microtitre dish-based biofilm assay, we were unable to detect significant differences between the WT strain and mutant H111-*bclACB* (lacking all three lectin genes), whereas the *cepR* mutant H111-R, which was included in these experiments as a control, was defective in biofilm formation (Fig. 3).

Since a defect in the microtitre dish-based biofilm assay was not observed for the H111-*bclACB* strain, we hypo-thesized that the function of the lectins could be masked by other adhesins. To test this hypothesis, we studied biofilm formation of a H111-*bclACB* mutant strain expressing *bapA* under a rhamnose-inducible promoter. Using this strain, we did observe a defect in biofilm formation when expression of BapA was reduced, suggesting a role of the lectins in surface colonization when BapA is limiting (Fig. 3). These results reinforce BapA as a major player in *B. cenocepacia* H111 biofilm formation and suggest that the lectins may be important under conditions when expression of BapA is repressed.

**Figure 5:**
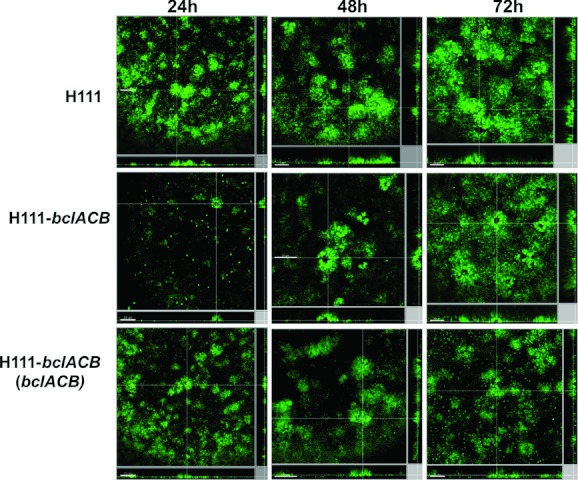
The *bclACB* operon influences biofilm development. Flow chambers were inoculated with the H111 wild type, the lectin mutant H111-*bclACB*, and the complemented lectin mutant H111-*bclACB* (*bclACB*). Biofilms were grown at 30°C in AB minimal medium supplemented with 0.3 mM glucose. Confocal laser scannig microscopy (CLSM) pictures were taken at 24, 48, and 72 h postinoculation. The larger central plots show the top view and the pictures in the right and lower frames show vertical sections through the biofilms.

We then used flow-through cells to analyze the progression of biofilm formation of the lectin-deficient mutant H111-*bclACB*. For that purpose, we genetically tagged the WT and mutant with the green fluorescent protein (GFP) and followed biofilm development over a period of 72 h. The lectin mutant H111-*bclACB* was found to be impaired in the initial colonization of the glass surface but then the cells aggregated into comparatively tall microcolonies, which were visible already 24 h postinoculum (Fig. 5). The colonies then developed into hollow volcano-like structures, in which the inner region remained uncolonized. COMSTAT analysis of 48-h-old biofilms showed significant differences in biomass, average thickness, and roughness when H111-*bclACB* was compared to the WT (Fig. S7). These structures, which were most evident after 72 h of biofilm development, then often converged at the upper part to form cupola-like structures whereas others remained open. Notably, in old biofilms of the WT strain, we also observed the appearance of hollows within the biofilm but these were generally smaller in size (96 h or later; see Fig. S8). Staining of WT biofilms with propidium iodide indicated that these hollows are the result of localized cell death (Fig. S8), as has been described previously for aged *P. aeruginosa* biofilms (Webb et al. 2003). In contrast to an aged WT biofilm, the hollows observed in an H111-*bclACB* biofilm did not contain dead cells (Fig. S8), suggesting that the formation of these structures is the result of an altered cellular aggregation behavior rather than a consequence of cell death.

The contribution of the different genes of the *bclACB* operon to biofilm development was studied in more detail. To this end, biofilm development was followed in the lectin mutant H111-*bclACB* complemented with different genes of the *bclACB* operon, including *bclACB*, *bclAC*, *bclA*, and *bclB*. Our results show that the biofilm defect of the H111-*bclACB* mutant could only be rescued when the entire *bclACB* operon was provided *in trans* (Fig. 5). Complementation of H111-*bclACB* with *bclA*, *bclAC*, or *bclB* resulted in biofilms displaying a developmental defect (Fig. S9). These defects were quantified by determining the ratio of the microcolony cavity height by the height of the microcolony, as previously described for *P. aeruginosa* biofilms (Ma et al. 2009). We found no statistically significant difference between the biofilm formed by H111-*bclACB* and the mutant complemented with either *bclA*, *bclAC*, or *bclB* (Fig. S9). Taken together, these results suggest that all three lectins may cooperate in biofilm structural development of *B. cenocepacia* H111.

**Figure 6:**
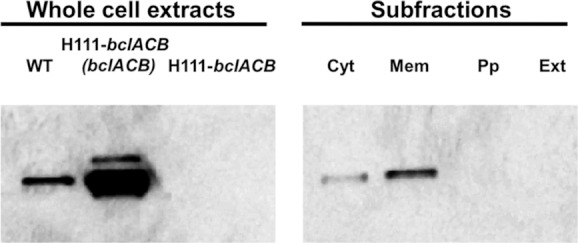
Subcellular localization of BclB in B. cenocepacia H111. Cells were grown on NB agar plates for 24 h at 37°C. Equivalent amounts of whole cell extracts from the wild type (WT), the lectin mutant (H111-*bclACB*), the complemented lectin mutant (H111-*bclACB(bclACB*)), and subfractions obtained from the supernatant of the cell suspension (Ext), the periplasm (Pp), cytoplasm (Cyt), and the membrane (Mem) were subjected to SDS-PAGE followed by immunoblotting using a BclB-specific antibody. The H111-*bclACB* strain served as negative control of the experiment.

### Lectin BclB is associated with the bacterial cell surface

The finding that the lectin-deficient strain H111-*bclACB* was impaired in the structural development of biofilms prompted us to investigate the possibility that the lectins are located on the bacterial cell surface. To this end, the subcellular localization of BclB was determined using cellular fractionation. Since the window of detection in liquid media was small and the protein was not detected after overnight incubation in liquid media (Fig. S6), we performed subcellular localization experiments from cells grown on NB media fortified with 1.5% agar. In this condition, BclB was easily detectable from 6 to 72 h of incubation, with an apparent maximum expression at 24 h of incubation (Fig. S10). As a control for proper fractionation, we measured the activity of glucose-6-phosphate dehydrogenase (G6PDH) in the different subfractions, which could only be detected in the cytoplasmic fraction (not shown). As evidenced by Western blotting, the large majority of the protein was found in the membrane fraction and only small amounts of BclB were observed in the cytoplasmic and none was detected in the periplasmic fraction (Fig. 6).

### FimA-dependent type 1 fimbriae are not essential for biofilm formation on abiotic surfaces

The third factor identified in our global analyses that may link QS and biofilm formation is a type 1 pilus. This surface appendage was previously shown to be important for the colonization of abiotic surfaces in *Escherichia coli* (Pratt and Kolter 1998). Specifically, our data show that the transcript levels of *fimA* (BCAL1677), which is homologous to the major subunit of type 1 fimbriae of *E. coli,* and the three succeeding genes BCAL1678-BCAL1680, which code for a putative chaperone-usher pathway (Holden et al. 2009), are downregulated in H111-R (Table 1; Fig. 1). Furthermore, we observed a decrease in the amounts of FimA in the EC fractions of mutants H111-R (−6.2-fold) and H111-I (−5.5-fold) as determined by iTRAQ analysis (Table S1). Interestingly, expression of BCAL1681, encoding a putative exporter protein, was also reduced in the H111-R transcriptome (−8.6-fold). These genes (BCAL1677–81) were confirmed to be an operon in RT-PCR experiments (Fig. S2). CepR-dependent expression of FimA was also confirmed by Western blotting employing antibodies directed against the protein (data not shown).

Measurements of a P*_fimA_-lacZ* fusion confirmed the microarray and proteome data since promoter activities were found to be reduced in the *cepR* mutant relative to the WT (Fig. 2). Previous work in *E. coli* has shown that expression of fimbrial genes is influenced by environmental factors (Schwan et al. 2002). To test this possibility, the effect of pH and osmolarity on the activity of the P*_fimA_*-*lacZ* fusion was determined (Fig. S11). We observed that the promoter showed maximum activity when cells were grown in a medium with low pH (5.0) and low osmolarity (17 mM NaCl) and importantly, QS-dependent regulation was also seen under these conditions (Fig. 2). In conclusion, these data show that in addition to the CepIR regulatory system, pH, osmolarity, and potentially other environmental factors are involved in the expression of the FimA-dependent type-1 pilus of *B. cenocepacia*.

We generated a defined *fimA* mutant, H111-*fimA*, as well as a mutant with a defect in the putative chaperon-usher transporter system (not shown). However, in neither mutant was the formation of static biofilms in microtitre dishes altered (Fig. 3) nor did we observe differences in biofilm structures in flow cell experiments when compared to WT biofilms (Fig. S12). We then tested whether the presence of BapA could mask the effect of FimA in static biofilms. However, and in contrast to what was observed for the BclACB lectins, no difference in biofilm production was observed in a *fimA* mutant with reduced levels of *bapA* expression (Fig. 3). Taken together, these results suggest that the CepR-controlled type-1 pilus of *B. cenocepacia* H111 is not essential for biofilm formation on abiotic surfaces under the conditions tested in this study.

## Discussion

In 1998, Davis et al. reported that a QS mutant of the opportunistic human pathogen *P. aeruginosa* was defective in biofilm formation (Davies et al. 1998). Specifically, it was shown that a mutant with an inactivated *lasI* gene (one of the two AHL synthase genes present in this organism) formed flat, densely packed, and undifferentiated biofilms whereas the WT formed biofilms consisting of typical mushroom-shaped microcolonies separated by water channels. However, subsequent studies revealed that the role of QS in biofilm formation of *P. aeruginosa* is less clearcut than initially anticipated: in studies using slightly changed experimental settings or hydrodynamic conditions, no significant differences between biofilms of the WT and those formed by QS-negative mutants were observed (Stoodley et al. 1999; Heydorn et al. 2002; Schaber et al. 2007), suggesting that different experimental settings have a strong influence on expression of QS-regulated genes. On the other hand, a number of QS-regulated functions, including the biosynthesis of rhamnolipids, the production of the biofilm matrix polysaccharide Pel, anaerobic denitrification, and *P. aeruginosa* quinolone signal (PQS)-dependent DNA release, have been identified that provide obvious links between biofilm development and cell–cell communication (for reviews, see Aguilar et al. 2009; de Kievit 2009).

A role for AHL-mediated QS in biofilm formation has also been demonstrated for *Aeromonas hydrophila* (Lynch et al. 2002), *Pseudomonas putida* (Steidle et al. 2002), and *Serratia marcescens* (Labbate et al. 2004; Rice et al. 2005). However, knowledge on the underlying molecular mechanisms linking QS and biofilm formation is scarce. Only in the case of the opportunistic pathogen *S. marcescens*, two QS-controlled genes, *bsmA* and *bsmB,* were identified and shown to be involved in adhesion and colonization of surfaces in the late stage of biofilm development (Labbate et al. 2004). The exact mechanisms of how BsmA and BsmB influence biofilm development, however, remain to be elucidated.

In *B. cenocepacia* H111, transposon mutagenesis was used to identify several genes required for biofilm formation (Huber et al. 2001, 2002). Although these studies provided clear evidence that the CepIR QS system is a major checkpoint for biofilm formation, the QS-controlled genes downstream of the regulatory cascade remained unidentified. In the present study, we have performed a detailed analysis of the *B. cenocepacia* H111 CepIR regulon using transcriptomics as well as proteomics. Of the various QS-regulated factors identified, we have chosen three functions that most obviously could link QS and biofilm formation: a chaperon-usher-type pilus, three lectins, and the large surface protein BapA.

### FimA pili

Pili are proteinaceous filaments on the bacterial surface that are employed in attachment and invasion, biofilm formation, cell motility, or protein and DNA transport across membranes. In this study, we demonstrate that expression of the FimA-like pilus BCAL1677 along with its putative assembly machinery (BCAL1678–BCAL1681) is positively CepR-regulated. BCAL1677 has previously been identified as a QS-regulated protein in strain H111 (Riedel et al. 2003). In a more recent study, O'Grady et al. (2009) have shown that expression of BCAL1677 in *B. cenocepacia* strain K56–2 is positively controlled by CepR and negatively regulated by the CciIR system, which is only present in *B. cenocepacia* strains containing the cenocepacia island (cci) found in highly transmissible ET12 strains (Baldwin et al. 2007). Moreover, our study shows that in addition to QS, the growth conditions significantly affect *fimA* expression. Similar to results reported for the uropathogenic *E. coli* strain NU149 (Schwan et al. 2002), we observed that pH as well as osmolarity strongly affected expression of *fimA*. However, in contrast to what was found in *E. coli,* expression of *fimA* in *B. cenocepacia* H111 was maximal when both pH and osmolarity were low. Although pili are very obvious and known factors required for the attachment of cells to surfaces, we observed that a BCAL1677-defective mutant strain formed biofilms that were virtually indistinguishable from the one of the WT. A plausible explanation for this observation is that pili often bind specifically to receptor sites, frequently present on biotic surfaces (De Greve et al. 2007). As the binding specificity of the BCAL1677 pilus is not known, we cannot rule out that this QS-regulated adhesin is required for the colonization of surfaces other than plastic or glass used in this study. Moreover, members of the Bcc are known to produce an array of different types of appendage pili (Goldstein et al. 1995) and the analysis of the J2315 genome indicated the presence of two additional chaperon-usher-type pili, two Flp-type pili, a type IV pilus, and the cable pilus (which is not present in strain H111, data not shown) (Holden et al. 2009). These different surface appendages may allow the organism to adhere to diverse surfaces in different habitats and may at the same time increase its unspecific adherence to glass or plastic, thereby masking the effect of inactivating BCAL1677.

### The large surface protein BapA

The *bapA* gene (BCAM2143) encodes a protein that belongs to a family of large surface proteins (Yousef and Espinosa-Urgel 2007; Reva and Tummler 2008). Many members of this protein family have been demonstrated to be involved in biofilm formation in various bacteria (e.g. Cucarella et al. 2001; Hinsa et al. 2003; Latasa et al. 2005; Tormo et al. 2005; Martinez-Gil et al. 2010). In a previous study, we have isolated and characterized *B. cenocepacia* H111 transposon insertion mutants that are defective in biofilm formation on abiotic surfaces (Huber et al. 2002). In two of the identified mutants, the transposon had interrupted BCAM2143 (*bapA*). In contrast to the WT, the *bapA* mutants grew in large but well-separated cell aggregates on the glass surface of the flow-through cells, giving rise to porous and disconnected biofilms. Using defined mutants, we were able to confirm the central importance of *bapA* for biofilm formation of *B. cenocepacia* H111 in this study. Large surface proteins are believed to be transported via type I secretion systems to the cell surface (Hinsa et al. 2003). We have provided evidence that *bapA* forms an operon with three downstream genes (BCAM2142–BCAM2140) encoding an ABC transporter (Fig. S2). Given that inactivation of the downstream genes resulted in the same biofilm defects as observed with the *bapA* mutant strain, it appears likely that BapA is secreted via this secretion system (Fig. S3). In support of this hypothesis, we also observed that a mCherry-BapA fusion protein was detected on the cell surface of the WT strain but not the *bcam2141* transporter mutant (Fig. 4). In *Pseudomonas fluorescens*, expression of LapA (also a large surface protein) was demonstrated to be regulated by a highly complex regulatory circuitry that is responsive to the intracellular levels of cyclic diguanosine monophosphate (c-di-GMP) as well as diadenosine tetraphosphate (Ap4A) (Newell et al. 2009; Monds et al. 2010). While additional regulatory mechanisms cannot be excluded at the moment, in the present study, we show for the first time that expression of a large surface protein is regulated by an AHL-dependent QS system. In fact, our data demonstrate that not only BapA but also the operon encoding the putative BapA secretion apparatus is positively regulated by the CepIR system in *B. cenocepacia* H111.

Previous work has provided evidence that the N-terminal region of BapA is a 22-kDa adhesin (named AdhA) that is associated with the cable pilus, on which it is distributed throughout the shaft (Sajjan et al. 2003; Urban et al. 2005). The AdhA adhesin has been implicated in binding to buccal epithelial cells (Sajjan et al. 2003) and lung explants from CF patients (Sajjan et al. 2000) using cytokeratin 13 (CK13), an intermediate filament that is part of the cell's cyto-skeleton, as a host cell receptor. Interestingly, CK13 expression is increased in CF airways, mainly in bronchiolar and respiratory epithelium (Sajjan et al. 2000), supporting the idea that cable pili may play an important role in the observed high transmissibility and pathogenicity of strains expressing this surface appendage (Mahenthiralingam et al. 1997; Clode et al. 2000). As mentioned above, sequence analysis of the H111 genome indicated that the strain does not possess the genes encoding the cable pilus. It is therefore tempting to speculate that BapA may serve different functions depending on the strain background. In this context, it is also noteworthy that expression of neither *bapA* nor the secretion system was found to be QS-regulated in *B. cenocepacia* strain K56-2, which is known to produce the cable pilus (Cheung et al. 2007).

### The BclACB lectins

Our transcriptome analysis identified that expression of the operon comprising the three genes BCAM0184 (*bclB*), BCAM0185 (*bclC*), and BCAM0186 (*bclA*), which code for lectins with PA-IIL domains, is CepR-induced (Fig. 2). This result is in full agreement with a recent transcriptome analysis of the CepIR QS system of *B. cenocepacia* K56–2 using the same custom microarray employed in this study, which revealed that transcription of *bclA* is positively controlled by the CepIR-system. Importantly, inspection of the *bclA* promoter region did not identify any obvious *lux* box-like elements as they have been found in the upstream region of the PA-IIL-encoding gene (Gilboa-Garber et al. 2000). This suggests that QS may indirectly affect *bclACB* expression, possibly via the *B. cenocepacia* orphan LuxR homolog CepR2 (Malott et al. 2009).

PA-IIL is a soluble lectin from *P. aeruginosa* that shows a strong affinity to fucose. Previous studies have not only demonstrated that PA-IIL is QS-regulated (Winzer et al. 2000) but also that a PA-IIL-deficient *P. aeruginosa* mutant is impaired in biofilm formation on glass slides when compared with the WT strain (Tielker et al. 2005). Moreover, using a transcriptomic approach it has been demonstrated that PA-IIL is differentially upregulated in developing biofilms (Hentzer et al. 2005). The shortest of the three *B. cenocepacia* genes (BCAM0186, *bclA*) has been analyzed in considerable detail (Lameignere et al. 2008, 2010). These investigations revealed that BclA forms homodimers with one binding site per monomer and displays a strict specificity for oligomannose-type oligosaccharides that are present on human glyco-proteins. While all three lectins share the PA-IIL-like C-terminal domain, BclB and BclC have additional N-terminal domains. A recent study demonstrated that the N-terminal domain of BclC is a tumor necrosis factor (TNF)-fold lectin with specificity for fucosylated glycans (Sulak et al. 2010). In other words, BclC is a chimeric protein consisting of two lectin domains with different sequences, folds, carbohydrate specificities, and quaternary arrangements. The N-terminal domain of BclB displays no similarity with known proteins.

In contrast to *P. aeruginosa* (Tielker et al. 2005), we did not observe that the lectin-deficient mutant H111-*bclACB* produced less biofilm mass on a plastic surface in a static biofilm assay. Only when expression of BapA was downregulated, an effect of the lectins on biofilm formation was evident (Fig. 3). Given that large surface proteins like BapA are thought to be associated with the outer membrane, it appears likely that it shields the lectins and we showed here that at least one of them, BclB, is also located at the cell surface (Fig. 6).

The structure of a lectin mutant biofilm is different from the one of the WT when biofilms were cultured on a glass surface in flow-through cells. Specifically, we observed hollow structures within the mutant biofilm matrix that were not the result of cell death. This change in biofilm morphology could only be restored to WT levels when complementing with the intact *bclACB* operon *in trans*, suggesting that the three lectins are not redundant in function and that all three are required for biofilm structural development. These results demonstrate that the lectins play an important role in biofilm structural development. It is tempting to speculate that surface-exposed BclACB lectins may mediate contact to neighboring cells within the biofilm or with the biofilm matrix. In this context, it is worth noting that it has been observed that in the presence of a suitable sugar ligand, BclA can form lattices or filaments that could potentially connect cells with each other or with the extracellular polysaccharides (EPS) of the biofilm (Lameignere et al. 2010). Although additional work will be required to unravel the biological functions of the three lectins in better detail, our data suggest that the lectins play an important role in the development of mature biofilms.

## Conclusions

Bacteria living in biofilms are embedded in a matrix composed of EC polysaccharides, proteins, and nucleic acids and therefore cell densities are obviously very high in these communities. Moreover, the biofilm matrix may also constitute a diffusion barrier for signal molecules, creating an environment that appears to be ideal for QS. In fact, several reports have demonstrated that QS occurs in biofilms and evidence has accumulated that, at least in some bacteria, QS plays an important role in biofilm formation. However, knowledge on the exact underlying molecular mechanisms is scarce. In the present study, we have demonstrated that of the various QS-regulated factors in *B. cenocepacia* H111, the large surface protein BapA has the greatest influence on biofilm formation on an abiotic surface, supporting previous reports on the pivotal role of members of this protein family for the formation of surface-associated aggregates (for a review, see Yousef and Espinosa-Urgel 2007). Furthermore, we show that a cluster of three Bcc lectins is required for biofilm structural development without affecting the total amount of biofilm formed. Work under progress aims at elucidating how BapA and the lectins possibly interact with other components of the biofilm matrix.

## Experimental Procedures

### Bacterial strains, plasmids, and growth conditions

Strains and plasmids used in this study are listed in Table S2. Unless otherwise stated, strains were grown aerobically at 37°C in modified Luria-Bertani (LB) broth (Andersen et al. 1998) or in “solution A and B” (AB) minimal medium (Clark and Maaløe 1967) supplemented with either 10 mM sodium citrate or 10 mM D-glucose. When needed, LB was buffered to pH 5 with 100 mM potassium phosphate. Anti-biotics were added as required at final concentrations of 100 μg/mL ampicillin, 50 μg/mL kanamycin, 10 μg/mL gentamicin, 100 μg/mL trimethoprim, 60 μg/mL chloramphenicol, and 25 μg/mL streptomycin. Growth was spectrophoto-metrically monitored by measurement of optical density at 600 nm.

### DNA manipulations, conjugative plasmid transfer, and nucleotide sequencing

All routine DNA manipulations were performed using standard methods (Sambrook et al. 1989). Plasmid DNA was isolated with a miniprep kit (Qiagen, Hilden, Germany), chromosomal DNA of *B. cenocepacia* strains was isolated by the sarkosyl-pronase method (Better et al. 1983). Triparental matings from *E. coli* to *B. cenocepacia* were performed with helper strains *E. coli* (pRK600) or *E.coli* (pRK2013) as described (Huber et al. 2001). Sequencing reactions were performed with the ABI 3730 DNA analyzer using the ABI BigDye® Terminator Cycle Sequencing kit (Applied Biosystems, Foster City, CA, USA).

### Mutagenesis and allelic replacement

Deletion mutants were generated by allelic replacement using a modified version of the Gateway cloning system (Carlier et al. 2009). Flanking regions of *bapA* were amplified by PCR using oligonucleotides CA52G_bapUPF, CA61_bapUPR, CA55G_bapDOR, and CA62_bapDOF (Table S3). Flanking regions of operon *bclACB* were amplified employing oligonucleotides lecUp-GW, lecUp-kan, lecDn-GW, and lecDn-kan (Table S3). A kanamycin cassette derived from plasmid pKD4 (Datsenko and Wanner 2000) was inserted between the flanking regions by means of overlap PCR reactions. The resulting PCR products were cloned into the Gateway Entry vector pDONR221 using the BP Enzyme II mix (Invitrogen, Carlsbad, CA, USA) and then transferred into the suicide vector pAUC40 using the LR Enzyme II mix (Invitrogen, Carlsbad, CA, USA). The resulting plasmids (pAUC40-bapA and pAUC40-bcl) were transferred to *B. cenocepacia* by triparental mating (see above), selecting for kanamycin resistance and streptomycin sensitivity. Allelic replacements were verified by PCR (Table S3). Complementation of the *bclACB* deletion mutant was performed by expressing different versions of the *bclACB* operon cloned in the vector pBBR1MCS-5. Genes were amplified by PCR using the oligonucleotides pairs CA150_bclACB_F and CA151_bclACB_R (for *bclACB*), CA150_bclACB_F and AS02_bclAC-R_BamHI (for *bclAC*), CA150_bclACB_F and AS03_bclA-R_BamHI (for *bclA*), CA180_bclB_F_HindIII and CA180_bclB_F_HindIII (for *bclB*), then cloned in pGEM-T (Invitrogen, Carlsbad, CA, USA). Fragments were excised with *BamH*I and *Hind*III (New England Biolabs, Beverly, MA, USA) and subcloned into the respective sites of pBBR1MCS-5. The resulting plasmids were introduced to the *bclACB* deletion mutant by triparental mating.

To generate an insertional mutant in *fimA*, a 301-bp internal fragment of BCAL1677 was amplified by PCR using oligonucleotides *fimA*_F and *fimA*_R (Table S3) and inserted as an *EcoR*I fragment into respective sites of plasmid pEX19Gm generating pNS1. The plasmid was transferred to *B. cenocepacia* by triparental mating as described selecting for gentamicin-resistant colonies. The integrity of the insertion was verified by PCR using oligonucleotides P*fimA*_F and pEXcheck_R (Table S3).

### Construction of transcriptional *lacZ* fusions

The upstream regions of *bclA*, *bapA*, and *fimA* were amplified by PCR using the oligonucleotides listed in Table S3. PCR fragments were cloned as *Xho*I/*Hind*III fragments into the respective sites of the promoter-probe vector pSU11 (Malott et al. 2009) generating plasmids P*_bclA_-lacZ*, P*_bapA_-lacZ*, and P*_fimA_-lacZ*. Plasmids were transferred to *B. cenocepacia* by triparental mating. ß-galactosidase activity of the reporter, normalized by cell growth, was measured as previously described (Stachel et al. 1985).

### RNA extraction and transcriptome analysis

Cells were grown to an OD_600_ of 2.5 in LB medium and RNA was extracted using the RiboPure™-Bacteria Kit (Ambion, Austin, TX, USA) as recommended by the manufacturer with the following modifications: to improve RNA yield, the lysate–ethanol mixture from two tubes of each sample was transferred to one filter cartridge; to remove trace amounts of genomic DNA samples were treated with DNase I (60 min, 37°C). Total RNA concentration and integrity was monitored using a ND-1000 NanoDrop spectrophotometer (Thermo Fisher Scientific, Wilmington, DE, USA) and employing agarose gel electrophoresis. Two-color microarray experiments were performed at the School of Biosciences at Cardiff University using Agilent 4-pack Bcc gene chips (Holden et al. 2009). The chip comprises 10,264 gene probes with 8741 sequences of *B. cenocepacia* strain J2315, 1070 gene sequences belonging to *B. cenocepacia* strain AU1054 and 387 gene sequences of *B. cenocepacia* HI2424. In total, three biological replicates of each of the analyzed strains were prepared. Total RNA (10–20 μg) was used for synthesis of cDNA. Labeling of first strand cDNA was performed according to the CyScribe Post-Labelling Kit Protocol (GE Healthcare, Munich, Germany). Following purification (CyScribe GFX Purification Kit, GE Healthcare, Munich, Germany), the cDNA was coupled with CyDye *N*-hydroxysuccinamide (NHS) ester. The CyDye-labeled cDNA was again purified (CyScribe Post-Labelling Kit, GE Healthcare, Munich, Germany). Transcriptome analyses were performed in collaboration with the CF foundation therapeutics. Microarray data were analyzed using the GeneSpring (v.7.3.1) software and further processed using the “Affimetrix FE” data normalization procedure recommended for Agilent arrays. Statistical analysis was carried out using analysis of variance (ANOVA) and the “Benjamini and Hochberg False Discovery Rate (BH_FDR) multiple testing correction.” Differently expressed genes that hybridized to array probes belonging to *B. cenocepacia* strain AU1054 (six CDSs), tRNA genes (19 CDSs), as well as intergenetic regions (21 CDSs) were excluded in this study. The entire microarray dataset has been deposited in the ArrayExpress database (http://www.ebi.ac.uk/arrayexpress) under the ID number E-MTAB-509.

### Protein extraction and analysis by iTRAQ

For comparative proteome analyses, 500 mL LB medium in 3l Erlenmeyer flasks were inoculated with precultures of the H111 WT, *cepR* mutant (H111-R), and *cepI* mutant (H111-I) and grown at 37°C under vigorous shaking (225 rpm). Strain H111-I was inoculated with or without the addition of 0.2 μM C_8_-HSL. Cells were harvested at an OD_600_ of 3.0. Following centrifugation (5000 rpm, 15 min), supernatants were separated from bacterial cell pellets and sterile filtered. Extracellular (EC) proteins were precipitated from sterile filtered culture supernatants with 15% trichloroacetic acid (TCA). Protein pellets were washed with acetone, dried at room-temperature (RT), and resuspended in 50 mM Tris-HCl, pH 7.5. Proteins were further purified by phenol extraction as described elsewhere (Riedel et al. 2003). The extraction of whole-cell (WC) proteins, followed by protein quantification, reduction, digestion, and labeling of the peptides with iTRAQ reagents was performed as described (Carranza et al. 2010). Four independent protein analyses were performed, in which the peptides were tagged with the different iTRAQ labels. The iTRAQ samples were separated into 27 fractions on a cation-exchange column (2.1 mm × 200 mm strong cation exchange (SCX)-column, PolySULPHOETHYL A, 5 μm, 300 Å, PolyLC, Columbia, NY) using the gradient solutions mobile phase A (10 mM KH_2_PO_4,_ 25% acetonitrile, pH 3) and phase B (10 mM KH_2_PO_4_, 25% acetonitrile, and 35 mM KCl, pH 3). Peptides were eluted at a flow rate of 0.3 mL/min over the following gradient: 10 min 100% mobile phase A, 40 min 0–50% mobile phase B, 10 min 100% mobile phase B. Fractions were pooled to four master fractions according to the SCX spectrum and purified using a C-18 column (Sep-Pak cartridge, Waters Corporation, Milford, MA, USA). Samples were further analyzed by matrix-assisted laser desorption/ionization time-of-flight/ time-of-flight (MALDI-TOF/TOF) mass spectrometry as described in detail in the Supporting Information.

### Purification of the BclB lectin and generation of anti-BclB antibodies

The BclB-encoding sequence (BCAM0184) was amplified by PCR-employing primers lecB3-F and lecB3-R_his (Table S3). The amplicon was inserted into pCR2.1 (Invitrogen, Carlsbad, CA, USA), excised from the vector and cloned into plasmid pET28a (Novagen, Madison, WI), generating a His_6_*-*BclB tag fusion protein. For protein overexpression, plasmid pET-HisBclB was transformed into *E. coli* BL21 (DE3) and gene expression was induced by the addition of 1 mM IPTG. His_6_-BclB was purified by column chromatography employing a Ni-IMAC (GE Healthcare, Munich, Germany) and a Superose column (Superose 12 10/300 GL, GE Healthcare, Munich, Germany) according to the instructions of the manufacturer's. Purified His_6_-BclB protein (1 mg) was used to raise rabbit polyclonal antibodies (Coring System Diagnostix GmbH, Germany).

### Cell fractionation

For cell fractionation experiments, we used the method described by Tielker et al. (2005) with some modifications. Bacterial cells were incubated on nutrient broth (NB) plates (3 g/L Bacto Peptone (BD, Sparks, MD, USA), 5 g/L meat extract (Oxoid, Basingstoke, Hampshire, England)), fortified with 1.5% agar at 37°C for 24 h. After this, cells were scraped off, resuspended in 1 mL 0.9% NaCl, and OD 600 nm was adjusted to 4.0. From this cell suspension, 600 μL were used for subfractionation. The cells were centrifuged at 3000 × *g*; the supernatant was centrifuged again for 5 min at 10,000 × *g* and used to determine the content of BclB in the super-natant of the cell suspension (Ext). The cell pellet was suspended in 240 μL of 100 mM Tris-HCl (pH 8) containing 20% (w/v) sucrose. After the addition of 240 μL of the same buffer containing 5 mM EDTA and 200 μg lysozyme, the sample was incubated for 2.5 h at 37°C (shaking at 40 rpm). Spheroplasts were collected by centrifugation at 10,000 × *g* for 20 min, and the supernatant was used as the peri-plasmic fraction (Pp). Spheroplasts were disrupted by sonication (Sonopuls HD 2200, Bandelin, Berlin, Germany) in 1 mL of 100 mM Tris-HCl (pH 8) (8 × 10 sec, power of 15–20%). After centrifugation for 5 min at 5000 × *g* to remove intact cells and cell debris, the total membrane fraction (Mem) was collected by centrifugation for 45 min at 13,000 × *g* and the supernatant was used as the cytoplasmic fraction (Cyt). The total membrane fraction was suspended in 1 mL of 100 mM Tris-HCl (pH 8). Fractions were then concentrated by TCA precipitation. To a 1 mL sample, 100 μL of 0.15% deoxycholic acid (DOC) was added. The tubes were vortexed and incubated at room temperature for 10 min. A total of 50 μL of 100% TCA were added, then the samples were vortexed and incubated on ice for 45 min. Samples were centrifuged at 4°C at 13,000 × *g* for 15 min. Supernatants were discarded and the pellets were washed twice with cold ethanol–diethyl ether (1:1). The pellets were dried and suspended directly in loading sample buffer.

### Protein analysis and Western blotting

Protein concentrations were determined with the Coomassie Plus protein assay reagent kit (Pierce, Rockford, IL) according to the manufacturer's instructions. For Western blot analysis, protein fractions (2 mg/mL) of WC proteins and EC proteins (TCA extraction as described before) of 50 mL cultures grown in LB medium were separated on a 15% SDS-PAGE gel and transferred to a polyvinylidene difluoride (PVDF) membrane (Amersham Hybond™-P, GE Healthcare, Munich, Germany). Membranes were incubated with anti-BclB antibodies and alkaline phosphatase-conjugated anti-rabbit immunoglobulin G (Sigma, Deisenhofen, Germany). Enzymatic activity was detected using the NBT/BCIP dye (Roche, Penzberg, Germany) according to the instructions of the manufacturer.

### Construction of a rhamnose-inducible *bapA* and mCherry-BapA translational fusion

Using the vector pSC200 (Ortega et al. 2007), the expression of *bapA* was engineered to be induced upon addition of rhamnose to the media. The vector pSC200 was first digested with *Nde*I (New England Biolabs, Beverly, MA, USA) and then blunt ended with Klenow enzyme (Promega, Madison, WI, USA). The first 600 bp of *bapA* were amplified by PCR using oligonucleotides CA148 and CA149 using Pfu polymerase (Promega, Madison, WI, USA) and then cloned into the blunt-ended vector pSC200. The resulting plasmid, in which expression of *bapA* is controlled by a rhamnose-inducible promoter, was transferred to the *B. cenocepacia* WT H111 by triparental mating and the exconjugants were plated on Pseudomonas Isolation Agar (PIA) plates supplemented with trimethoprim (100 μg/mL). A chromosomal in-frame translational fusion of the gene coding for the red fluorescent protein mCherry and the N-terminus of BapA was generated as follows: the vector pSC200 was first digested with *Nde*I (New England Biolabs, Beverly, MA, USA) and then blunt ended with Klenow enzyme (Promega, Madison, WI, USA). *mCherry* was amplified by PCR using the oligonucleotides CA118 and CA133_*Xba*I using Pfu polymerase (Promega, Madison, WI, USA) and then cloned into the blunt-ended vector pSC200 generating plasmid pSC200-mCherry. Subsequently, a 600-bp DNA fragment of the 5′ region of *bapA* (containing the predicted starting codon) was amplified using the oligonucleotides CA134_*Xba*I and CA117_*Xba*I and cloned into pGEM-T (Promega, Madison, MA, USA). The *bapA* fragment was then digested with *Xba*I (underlined in the oligonucleotide sequences) and cloned into the corresponding *Xba*I site of pSC200-mCherry, generating plasmid pCA*mCherry-bapA*. The resulting plasmid was transferred to the *B. cenocepacia* WT H111 as well as the transporter mutant H111-*bcam2141* by triparental mating and the exconjugants were plated on PIA plates supplemented with trimethoprim (100 μg/mL). The correct insertion in the *B. cenocepacia* chromosome was confirmed by PCR. To determine the subcellular localization of the mCherry-BapA fusion protein, cells were grown on M9 medium fortified with 1.5% agar and supplemented with 0.5% (w/v) rhamnose as sole carbon source. Following overnight incubation, cells were resuspended in 5 mL phosphate-buffered saline (PBS) (170 mM NaCl, 10 mM phosphate, 3 mM KCl, pH 7.4), fixed with 4% paraformaldehyde in PBS for 10 min, and washed three times with PBS. Microscopic inspection and image acquisition were performed on a Leica DM6000B microscope (Wetzlar, Germany) equipped with a 100 × 1.3 oil objective. Data were analyzed with the Leica Application Suite (Mannheim, Germany) and the Imaris software package (Bitplane, Zurich, Switzerland). Images were prepared for publication using Photoshop CS (Adobe Systems Incorporated, San Jose, CA, USA) and Powerpoint (Microsoft Corporation, Redmond, WA, USA) software.

### Analyses of biofilm formation under static and dynamic conditions

Biofilm formation in polystyrene microtitre plates (Sarstedt, Newton, MA) was assayed as described previously (O'Toole and Kolter 1998; Huber et al. 2001). Biofilms were cultured in AB minimal media supplemented with either 10 mM citrate or 10 mM D-glucose and incubated at 30°C for 48 h. To monitor biofilm formation under dynamic conditions, strains were tagged with GFP by introducing plasmid pBAH7 or pBAH8 (Table S2). For complemented strains, cells were stained with 3.34 μM SYTO-9 (Invitrogen, Carlsbad, CA, USA). Biofilms were cultured in artificial flow cells supplied with AB medium containing 0.3 mM glucose at 30°C. Dead cells were visualized by adding 0.5 μM propidium iodide (Fluka, Buchs, Switzerland) in AB medium. The biofilm system was assembled as previously described (Christensen et al. 1999). Medium flow was kept at a constant rate of 0.5 mm s^−1^ by using a Watson-Marlow 205S peristaltic pump. Biofilms were inspected microscopically with a confocal laser scanning microscope (Leica, DM 5500 Q, Wetzlar, Germany) equipped with a 40 × 1.15 oil objective. Data were analyzed with Leica Application Suite (Mannheim, Germany) and the Imaris software package (Bitplane, Zurich, Switzerland). Images were prepared for publication using Photoshop CS (Adobe Systems Incorporated, San Jose, CA, USA) and Powerpoint (Microsoft Corporation, Redmond, WA, USA) software.

## Supporting information

**Figure S1.** Transcription of *aidA* is stringently CepR-regulated.

**Figure S2.** Reverse-transcriptase PCR (RT-PCR) showing that the loci under study are organized as operons in *B. cenocepacia* H111.

**Figure S3.** The ABC transporter located downstream of *bapA* (BCAM2140–BCAM2142) is necessary for biofilm formation on a polystyrene surface.

**Figure S4.** Levels of *bapA* expression influence biofilm formation on a polystyrene surface.

**Figure S5.** H111-*bapA* is impaired in biofilm development.

**Figure S6.** Expression of the BclB lectin is controlled by the CepIR QS system.

**Figure S7.** COMSTAT comparison of H111 and H111-*bclACB*.

**Figure S8.** Cell death contributes to the formation of hollow colonies in biofilms of the wild type H111 but not of the mutant H111-*bclACB*.

**Figure S9.** Complementation of H111-*bclACB*.

**Figure S10.** Temporal expression of BclB on agar plates.

**Figure S11.** Expression of *fimA* as a function of pH and osmolarity.

**Figure S12.** Inactivation of *fimA* does not affect biofilm structure.

**Table S1.** QS-regulated proteins that are more than twofold differentially expressed in *cepI* mutant (H111-I) and/or *cepR* mutant (H111-R).

**Table S2**. Strains and plasmids used in this study.

**Table S3**. Oligonucleotide primers used in this study.

**Table S4**. Genes upregulated by ≥3 fold in the H111-R transcriptome versus H111 WT.
