# Supplementary material for: Identification of functions linking quorum sensing with biofilm formation in Burkholderia cenocepacia H111
Source: Microbiologyopen. 2012 Jun;1(2):225–42. doi: 10.1002/mbo3.24 (PMC3426421; doi:10.1002/mbo3.24)
Supplement: Supplementary file 1 [file mbo30001-0225-SD1.pdf]

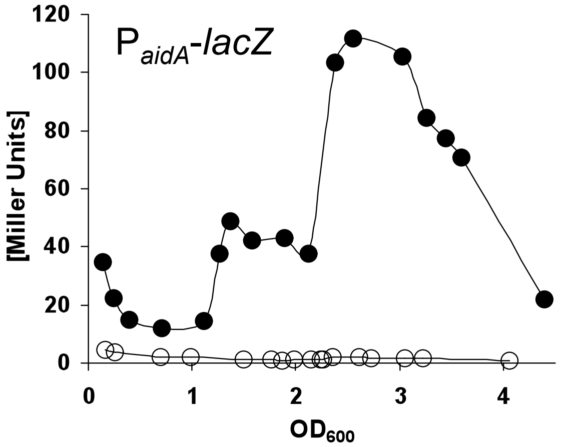

Fig. S1. **Transcription of *aidA* is stringently CepR-regulated.** β-galactosidase activities of a P_{aidA}-*lacZ* transcriptional fusion were determined in the parent *B. cenocepacia* H111 (filled symbols) and the *cepR* mutant H111-R (open symbols) in LB-medium throughout the growth. Transcription of *aidA* (BAS0293) was found to be maximal at an OD_{600} of approximately 2.5.

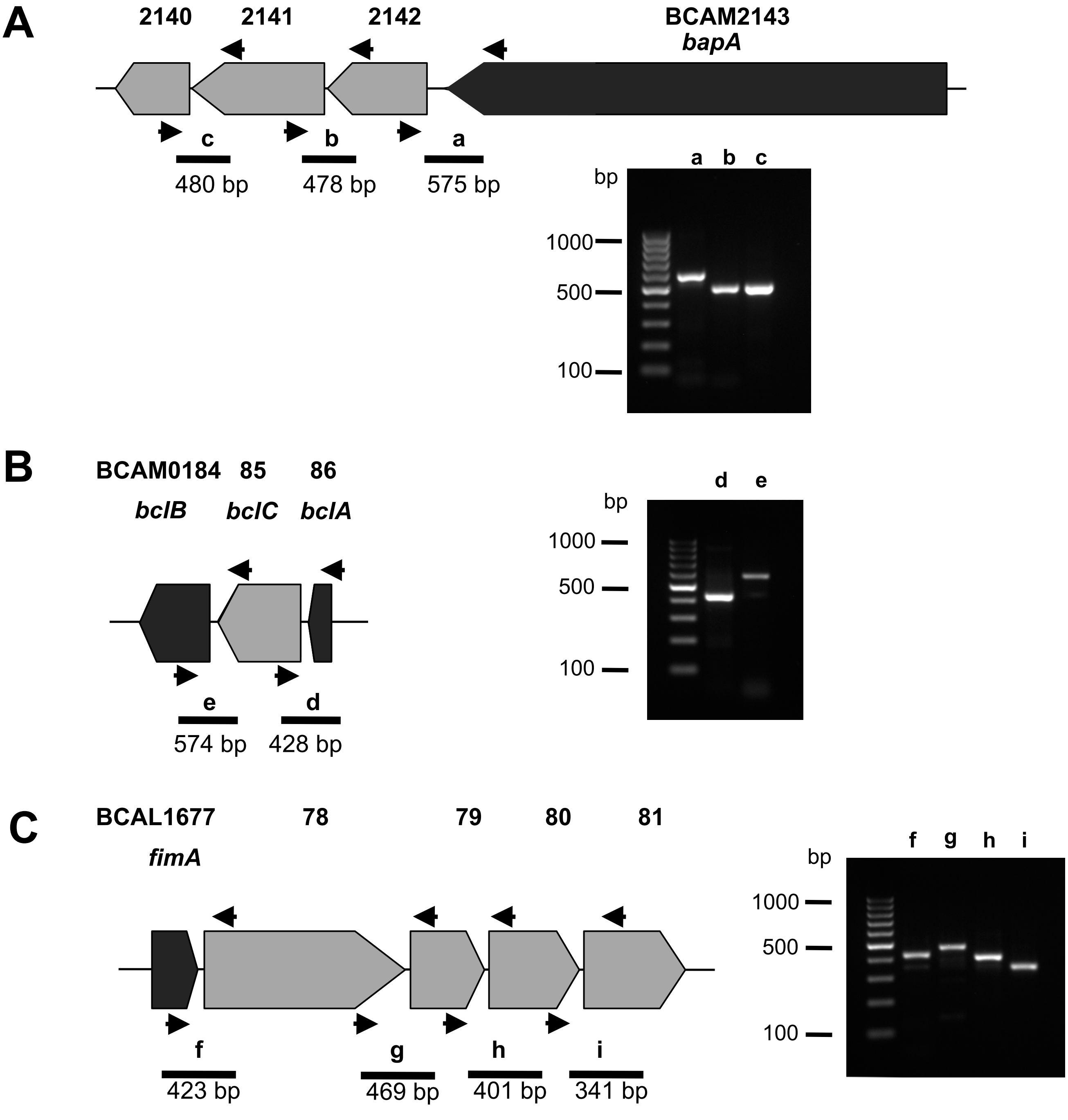

Fig. S2. Reverse-transcriptase PCR (RT-PCR) showing that the loci under study are organized as operons in *B. cenocepacia* H111. A, *bapA*; **B**, *bclACB*, **C**, *fimA*. Arrows indicate approximate position of the oligos used in the PCR reaction (left side) with cDNA (10ng) as template. Letters indicate the expected amplicon size, visualized in a 1.5% agarose gel (rigth images). No product was obtained when the RNA preparation was tested in a control PCR reaction (RT minus control, not shown). Pairs of primers used in the PCR reaction were CA168/169(a), CA170/171(b), CA172/173(c), CA154/155(d), CA156/157(e), CA174/175(f), CA176/177(g), CA178/179(h) and CA182/183(i).

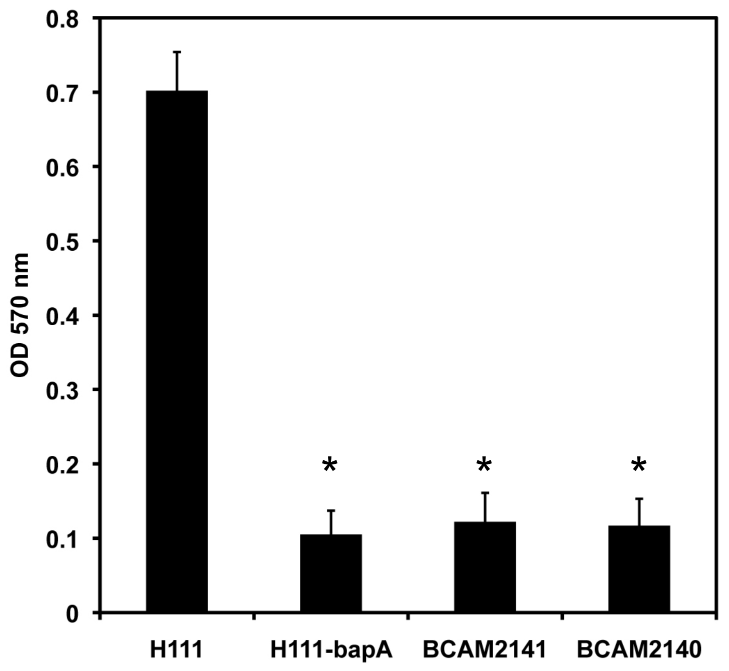

Fig. S3. The ABC transporter located downstream of *bapA* (BCAM2140 to BCAM2142) is necessary for biofilm formation on a polystyrene surface. Biofilms were grown for 48h in AB medium supplemented with 10 mM citrate and stained with crystal violet. *bcam2141* (BCAM2141::pEX-Gm) and *bcam2140* (BCAM2140::pEXGm) show a similar reduction in biofilm formation as H111-*bapA.* Asterisk indicates significant difference in biofilms of mutant strains when compared to H111 (t-test, P<0.01). Error bars indicate SEM, n=3.

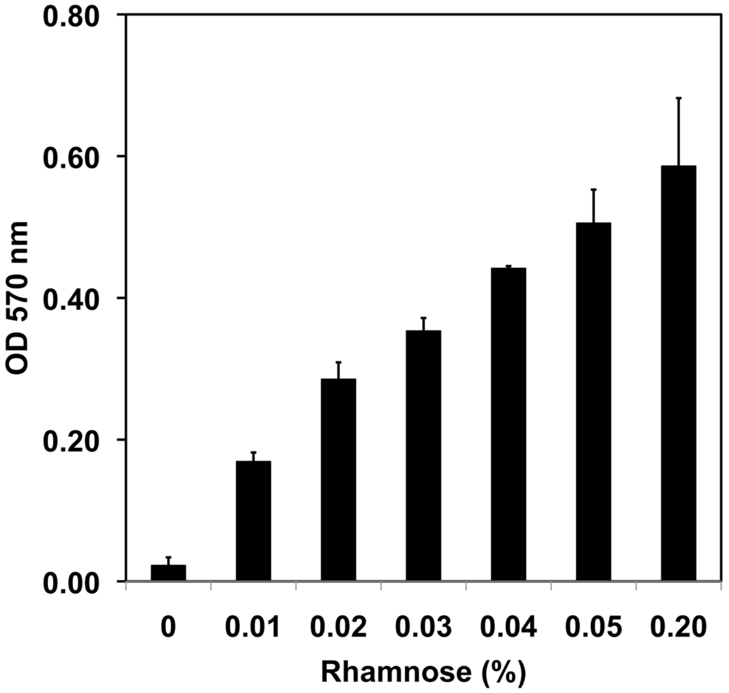

Fig S4. Levels of *bapA* expression influence biofilm formation on a polystyrene surface. Expression of *bapA* was controlled using a rhamnose-inducible promoter at the concentrations depicted in the figure. Bars are SEM, n=3.

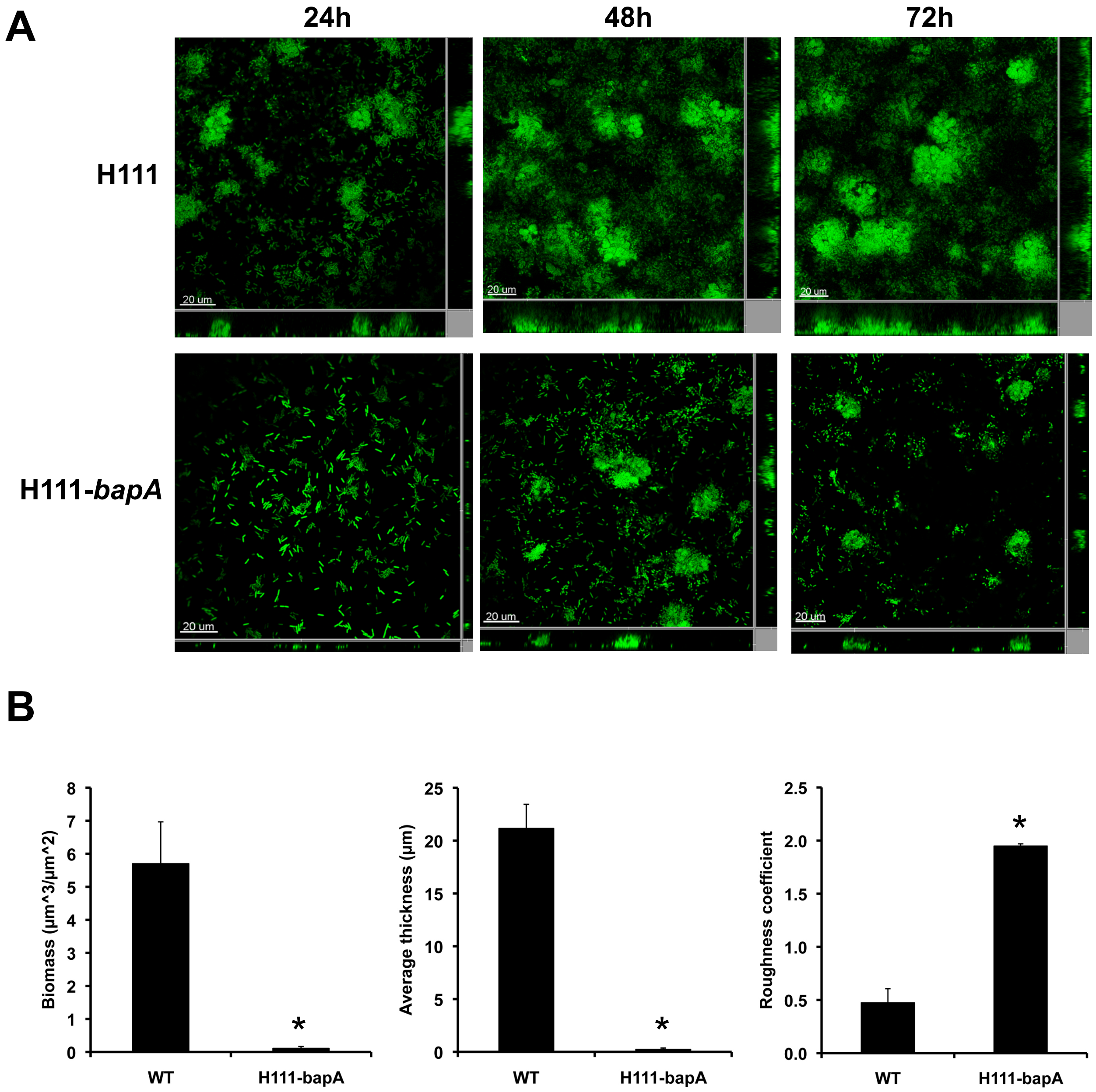

Fig S5. H111-*bapA* is impaired in biofilm development. A, flow chambers were inoculated with *gfp*-tagged derivatives of the H111 wild type or mutant H111-*bapA*. Biofilms were grown at 30°C in AB minimal medium supplemented with 0.3 mM glucose. CLSM pictures were taken at 24h, 48h and 72h post-inoculation. The larger central plots show the top view and the pictures in the right and lower frames show vertical sections through the biofilms. **B**, COMSTAT analysis of 48h biofilms showed significant differences in biomass (t-test, $P<0.005$), Average thickness (t-test, $P<0.001$) and roughness coefficient (t-test, $P<0.001$). $n=8$.

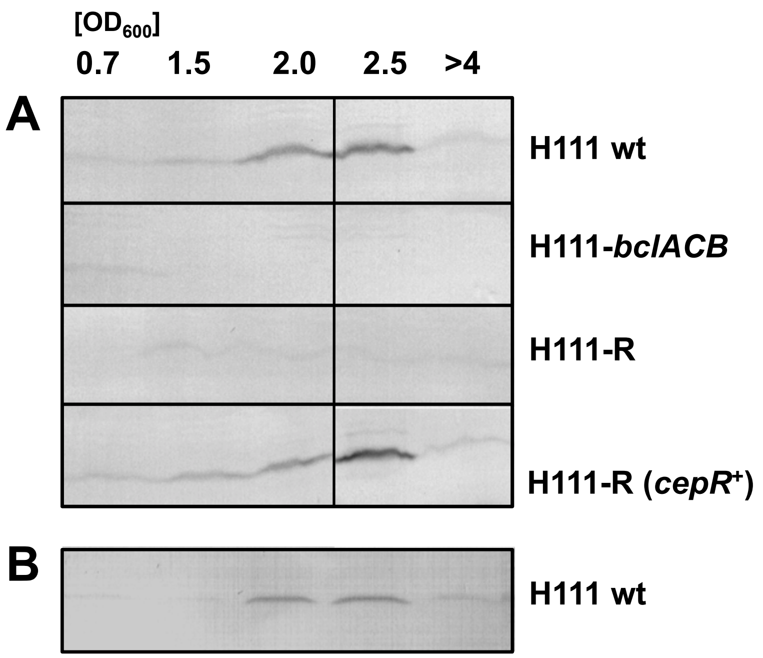

Fig. S6. Expression of the BclB lectin is controlled by the CepIR QS system. The *B. cenocepacia* H111 wild type (wt), mutant H111-*bclACB*, *cepR* mutant H111-R, and the complemented mutant H111-R (*cepR*$^{+}$) were grown in liquid LB-medium with agitation. Cells were harvested along the growth curve over a period of 24h. Whole cell proteins **(A)** were extracted from cell pellets, which were resuspended in buffer to an OD_{600} of 1 for sample normalization. Extracellular proteins **(B)** were extracted from the supernatants of the wild type samples in **A**. BclB was visualized by immunoblotting with polyclonal antibodies directed against the protein.

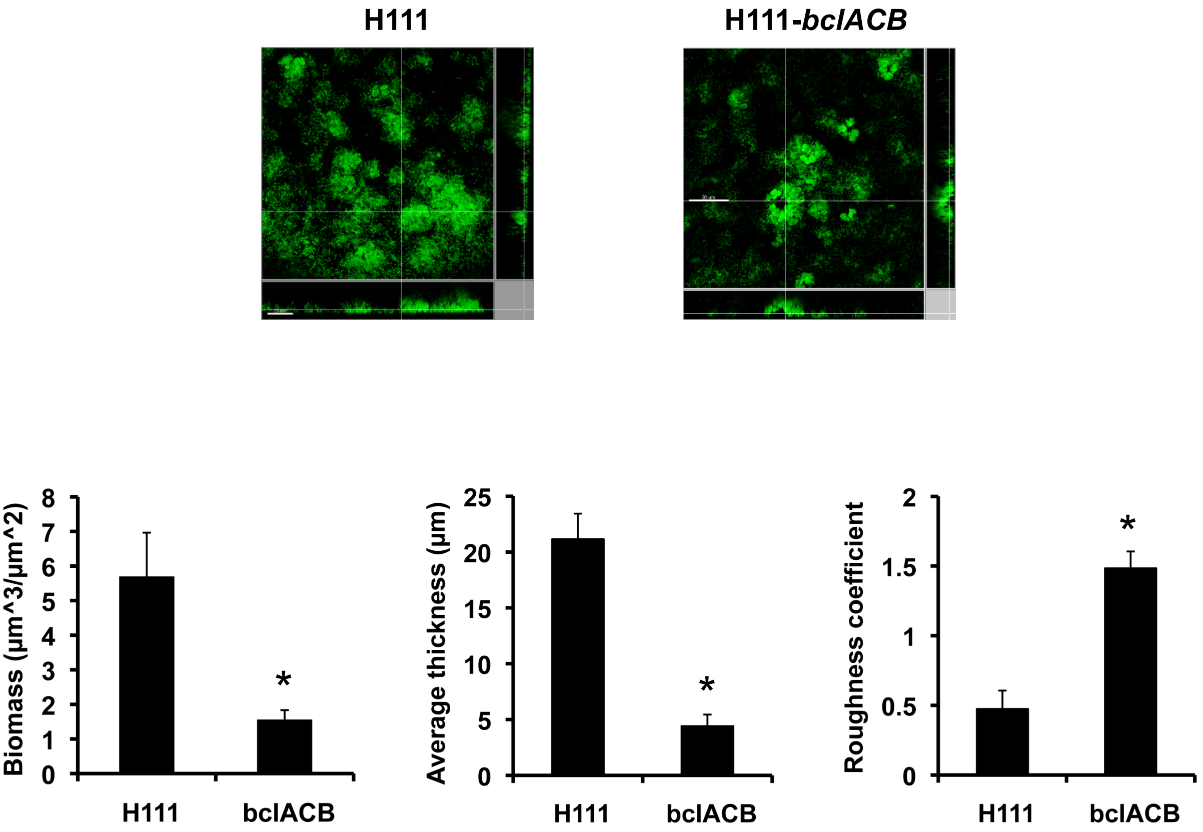

Fig S7. COMSTAT comparison of H111 and H111-*bclACB*. Biofilms of H111 and H111-*bclACB* analysed using COMSTAT at 48h of development. Asterisk indicate significant differences observed in biomass (t-test, $P<0.05$), average thickness (t-test, $P<0.001$) and roughness (t-test, $P<0.001$). Error bars indicate SEM, n=8.

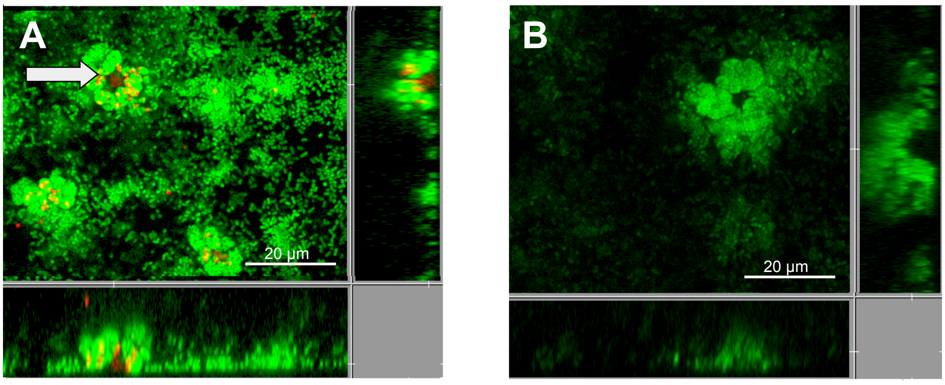

Fig. S8. Cell death contributes to the formation of hollow colonies in biofilms of the wild type H111 but not of the mutant H111-*bclACB*. (**A**) To visualize bacterial death, biofilms of *gfp*-tagged cells (green) of the *B. cenocepacia* H111 parent and mutant H111-*bclACB* were stained with propidium iodide (red). The arrow depicts dead cells (only dead cells are stained by propidium iodide), visible in the centres of microcolonies formed by the H111 wild type 96h post-inoculation. (**B**) Hollow microcolonies were observed already in 48h old biofilms of the *bcl* mutant H111-*bclACB*, but no dead cells were detected within the microcolonies.

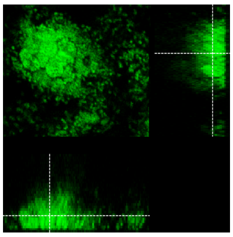

Strain	Height of cavity (b) / Height of Microcolony (a)
**H111-*bclACB***	**0.481 ± 0.014**
**H111-*bclACB*(*bclAC*)**	**0.496 ± 0.035**
**H111-*bclACB*(*bclA*)**	**0.476 ± 0.032**
**H111-*bclACB*(*bclB*)**	**0.532 ± 0.037**

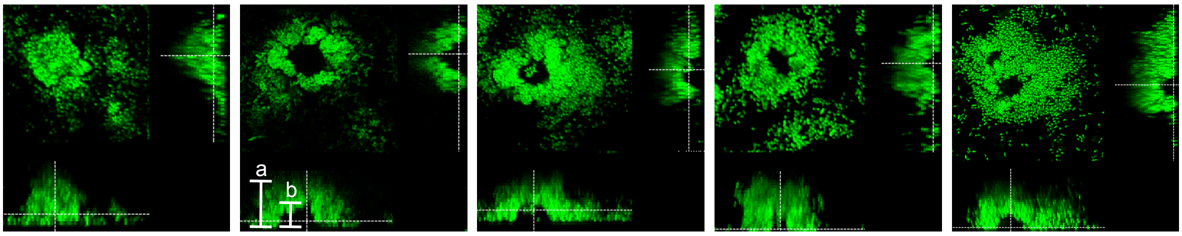

Fig S9. Complementation of H111-*bclACB*. The complementation of H111-*bclACB* with different components of the *bclACB* operon was evaluated by calculating the ratio of the height of the microcolony (b, depicted by a white bar) by the height of the whole microcolony (a). Data was obtained from independent microcolonies and no statistical difference was observed when H111-*bclACB* (28 microcolonies) was compared to H111-*bclACB*(*bclAC*) (t-test, P=0.71, n=15), H111-*bclACB*(*bclA*) (t-test, P=0.8, n=12), or *bclACB*(*bclB*) (t-test, P=0.24, n=7). No detectable cavities were found in H111 or in H111-*bclACB*(*bclACB*).

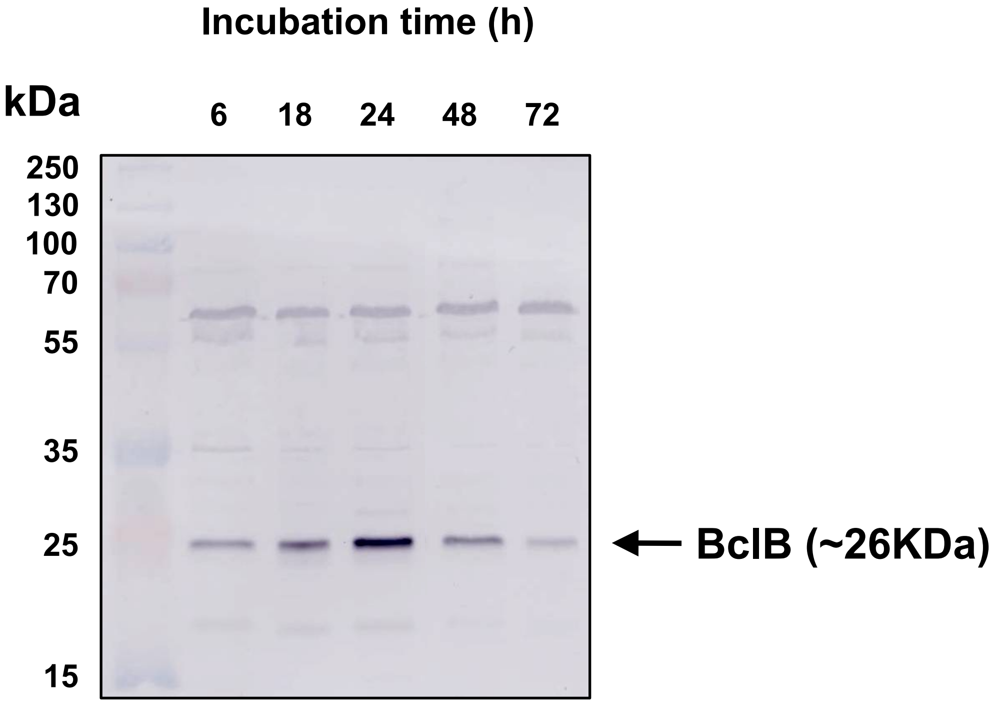

Fig. S10. Temporal expression of BclB on agar plates. *B. cenocepacia* H111 was inoculated on NB plates fortified with 1.5% agar at 37°C and cells were harvested at 6, 18, 24, 48 or 72h of growth. Cells were resuspended in 0.9% NaCl and OD 600nm was adjusted to 2.0. Equal volumes of each time-point were separated on a 12% SDS-PAGE gel and transferred to a polyvinylidene difluoride (PVDF) membrane (Amersham Hybond^{TM}-P, GE-Healthcare). Membranes were incubated with anti-BclB antibodies and alkaline phosphatase-conjugated anti-rabbit immunoglobulin G (Sigma, Deisenhofen, Germany). Enzymatic activity was detected using the NBT/BCIP dye (Roche, Penzberg, Germany) according to the instructions of the manufacturer. The maximal signal for BclB was detected after 24h of incubation.

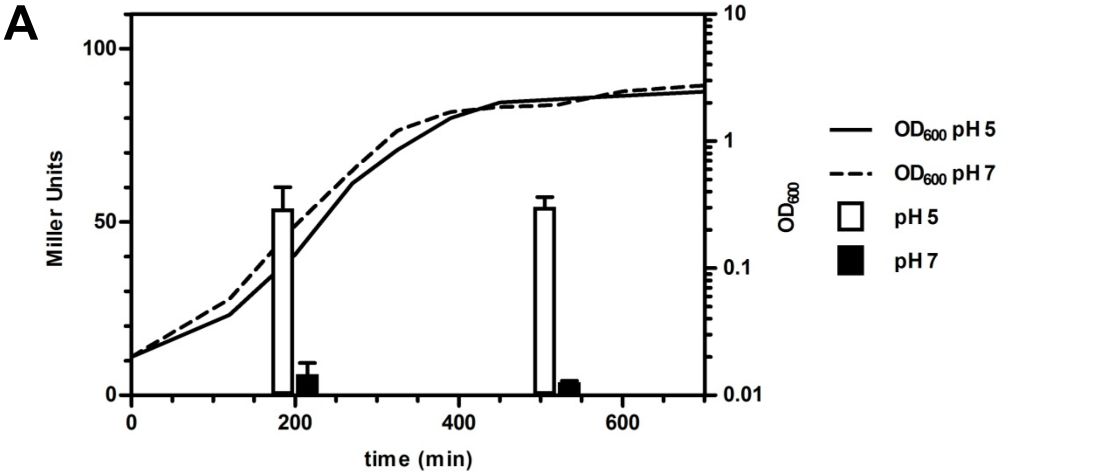

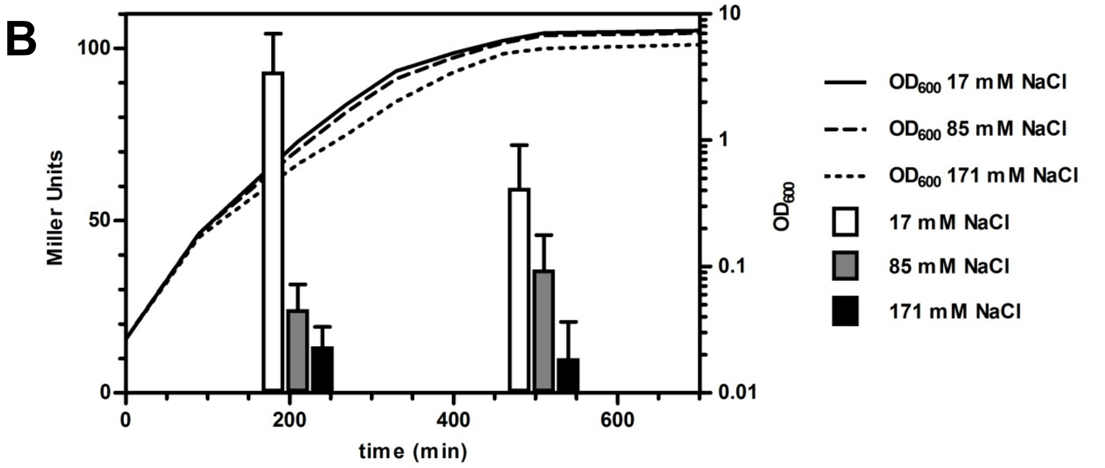

Figure S11. Expression of *fimA* as a function of pH and osmolarity. ß-galactosidase activitiy of the *fimA* promoter region fused to *lacZ*, assayed in *B. cenocepacia* H111 at mid-log or early stationary phases of growth. **A,** LB Lennox buffered to pH 5 or 7. **B,** LB buffered to pH 5 and containing NaCl at 17mM (1 g/L), 85 mM (5 g/L) or 171 mM (10 g/L). Values are means of n = 3. Error bars indicate SD.

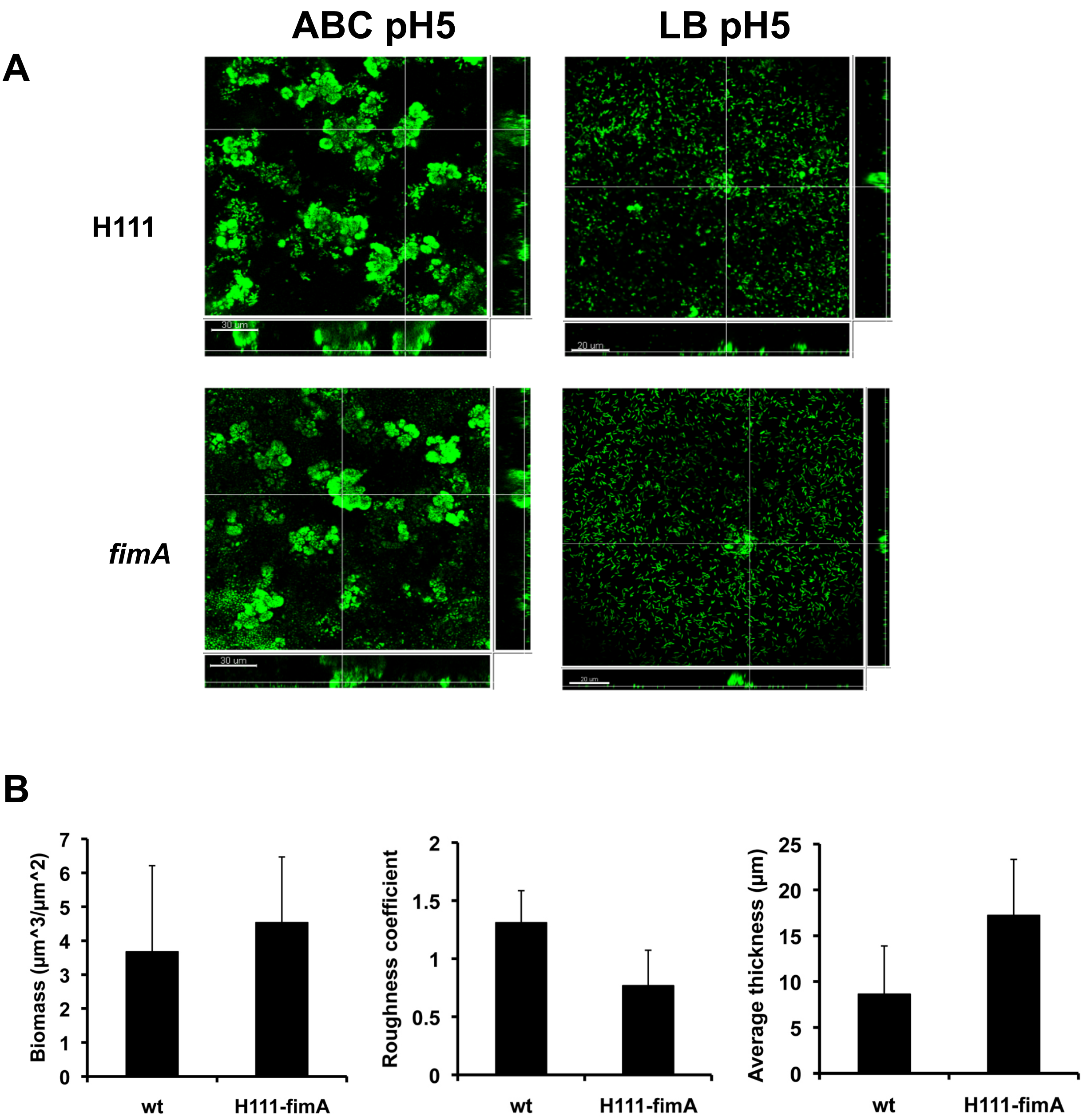

Figure S12. Inactivation of *fimA* does not affect biofilm structure. Flow chambers were inoculated with *gfp*-tagged derivatives of the H111 wild type or mutant H111-*fimA*. **A,** Biofilms were grown at 30°C in AB minimal medium buffered to pH5 and supplemented with 10 mM citrate or in 20 times diluted LB buffered to pH5. CLSM pictures were taken 72h post-inoculation. The larger central plots show the top view and the pictures in the right and lower frames show vertical sections through the biofilms. **B,** COMSTAT analysis of biofilms grown in ABC pH5, showing no significant differences between wt and H111-*fimA* biofilms in biomass (t-test, P=0.79), roughness (t-test, P=0.32) or thickness (t-test, P=0.22). n=10. Error bars indicate SEM.
