# Supplementary material for: Identification of functions linking quorum sensing with biofilm formation in Burkholderia cenocepacia H111
Source: Microbiologyopen. 2012 Jun;1(2):225–42. doi: 10.1002/mbo3.24 (PMC3426421; doi:10.1002/mbo3.24)
Supplement: Supplementary file 2 [file mbo30001-0225-SD2.pdf]

Supporting Methods.

Nano-LC separation and MALDI target spotting of tryptic peptides

Samples were further analysed by nano-liquid chromatography coupled to matrix-assisted laser desorption/ionization time-of flight/time-of-flight tandem mass spectrometry (nano-LC-MALDI-TOF/TOF MS). Peptide separation was performed on an Ultimate chromatography system (Dionex-LC Packings, Sunnyvale, CA) equipped with a Probot MALDI spotting device. Samples (5 µl) were injected by using a Famos autosampler (Dionex-LC Packings) and loaded directly onto a 75 µm x 150 mm reversed-phase column (PepMap 100, 3 mm; Dionex-LC Packings). Peptides were eluted at a flow rate of 300 nl/min by using the following gradient: 0-10 min, 0% solvent B; 10-105 min, 0-50% solvent B; and 105-115 min, 50-100% solvent B. Solvent A contained 0.1% TFA in 95:5 water/acetonitrile, and solvent B contained 0.1% TFA in 20:80 water/acetonitrile. For MALDI analysis, the column effluent was directly mixed with MALDI matrix (3 mg/ml a-cyano-4-hydroxycinnamic acid in 70 % acetonitrile/0.1 % TFA) at a flow rate of 1.1 µl/min via a µ-Tee fitting. Fractions were automatically deposited every 10 s onto a MALDI target plate (Applied Biosystems, Toronto, Canada) using a Probot micro fraction collector. A total of 416 spots were collected from each HPLC run.

MALDI-TOF/TOF mass spectrometry

MALDI plates were analysed on a 4800 MALDI TOF/TOF system (Applied Biosystems, Toronto, Canada) equipped with a Nd:YAG laser operating at 200 Hz. All mass spectra were recorded in positive reflector mode and generated by accumulating data from 800

laser shots. First, MS spectra were recorded from peptide standards on each of the six calibration spots, and the default calibration parameters were updated. Second, MS spectra were recorded for all sample spots on the MALDI target plate (416 spots per sample, 4 samples per plate). The MS spectra were recalibrated internally based on the ion signal of neurotensin peptide (Sigma, Buchs, Switzerland). Spectral peaks that met the threshold criteria and were not on the exclusion list were included in the acquisition list for the MS/MS spectra. The following threshold criteria and settings were used: Mass range: 800 to 4000 Da; minimum signal-to-noise (S/N) for MS/MS acquisition: 100; maximum number of peaks/spot: 8. Peptide CID was performed at a collision energy of 1 kV and a collision gas pressure of approximately 2.5 x 10-6 Torr. During MS/MS data acquisition, a method with a stop condition was used. In this method, a minimum of 1000 shots (20 sub-spectra accumulated from 50 laser shots each) and a maximum of 2000 shots (40 sub-spectra) were allowed for each spectrum. The accumulation of additional laser shots was halted whenever at least 6 ion signals with a S/N of at least 60 were present in the accumulated MS/MS spectrum, in the region above m/z 200.

Protein identification and relative quantification

GPS (Global Proteomics Server) Explorer Software (Applied Biosystems, Foster City, USA) was used for submitting data acquired with the MALDI-TOF/TOF mass spectrometer for database searching. The MS and MS/MS data were searched using Mascot version 2.1.0 (Matrix Science, London, UK) as the search engine (Perkins et al., 1999). The following search settings were used: maximum missed cleavages: 1; maximum number of signals per spectrum: 55; peptide mass tolerance: 35 ppm MS/MS

tolerance: 0.2 or 0.25 Da. ITRAQ labelling of lysine and of the N-terminal amino group of peptides and methyl methanthiosulfonate (MMTS) derivatization of cysteine were specified as fixed modifications. All searches were performed against two databases (p142_burkhold, 148894 entries and p142_ceno_J2315, 7525 entries) comprising annotated proteins of various *Burkholderia* species (*B. cenocepacia* J2315, *B. cenocepacia* AU 1054, *B. cenocepacia* HI2424, *B. cepacia* AMMD, *B.* sp. 383, *B. mallei* ATCC 23344, *B. mallei* NCTC 10229, *B. mallei* NCTC 10247, *B. mallei* SAVP1, *B. pseudomallei* 1106a, *B. pseudomallei* 1710b, *B. pseudomallei* 668, *B. pseudomallei* 305, *B. pseudomallei* K96243, *B. thailandensis* E264, *B. vietnamiensis* G4, *B. xenovorans* LB400, *B. ambifaria* MC40-6, *B. cenocepacia* MC0-3, *B. multivorans* ATCC 17616, *B. phymatum* STM815, *B. phytofirmans* PsJN) and proteins of the closest relative of H111, *B. cenocepacia* J2315, respectively. Moreover, common contaminants such as trypsin and keratin were added to both of the databases to avoid false positive identification of proteins.

The ratio of peak areas between iTRAQ reporter ions 114, 115, 116 and 117 (analyses I and II) and reporter ions 117, 116, 115 and 114 (analyses III and IV) was used to determine the relative abundance of proteins in each protein sample. For normalization the ratio of each protein sample from iTRAQ analyses I or II and III or IV were multiplied. The root of the product was then extracted and further referred to as "normalized". The mean, standard deviation, and p-values to estimate statistical significance of protein quantification were calculated by the Mascot software. Proteins were assigned as QS-regulated when the regulation factors were higher than 2 in both

independent analyses of either EC and/or WC proteins of cepR and or cepI mutant and the corresponding p-values were below 0.05.

Glucose-6-phosphate-dehydrogenase assay.

The test solution contained 10 mM glucose-6-phosphate (Sigma), 0.5 mM NADP (Sigma), 200 mM Tris-HCl (pH 8.5) and 10 mM 2-mercaptoethanol (AppliChem). The cellular subfractions (obtained as described in the Methods section) were tested for Glucose-6-phosphate-dehydrogenase (G6PDH) activity by mixing 100µl of each fraction with 900µl of test solution. The increase in the optical density (OD 340 nm) was monitored spectrophotometrically (Ultrospec 2100 pro, Amersham Biosciences) at 25°C for 10min.
