# Supplementary figures and images for: Identification of functions linking quorum sensing with biofilm formation in Burkholderia cenocepacia H111
Source: Microbiologyopen. 2012 Jun;1(2):225–42. doi: 10.1002/mbo3.24 (PMC3426421; doi:10.1002/mbo3.24)

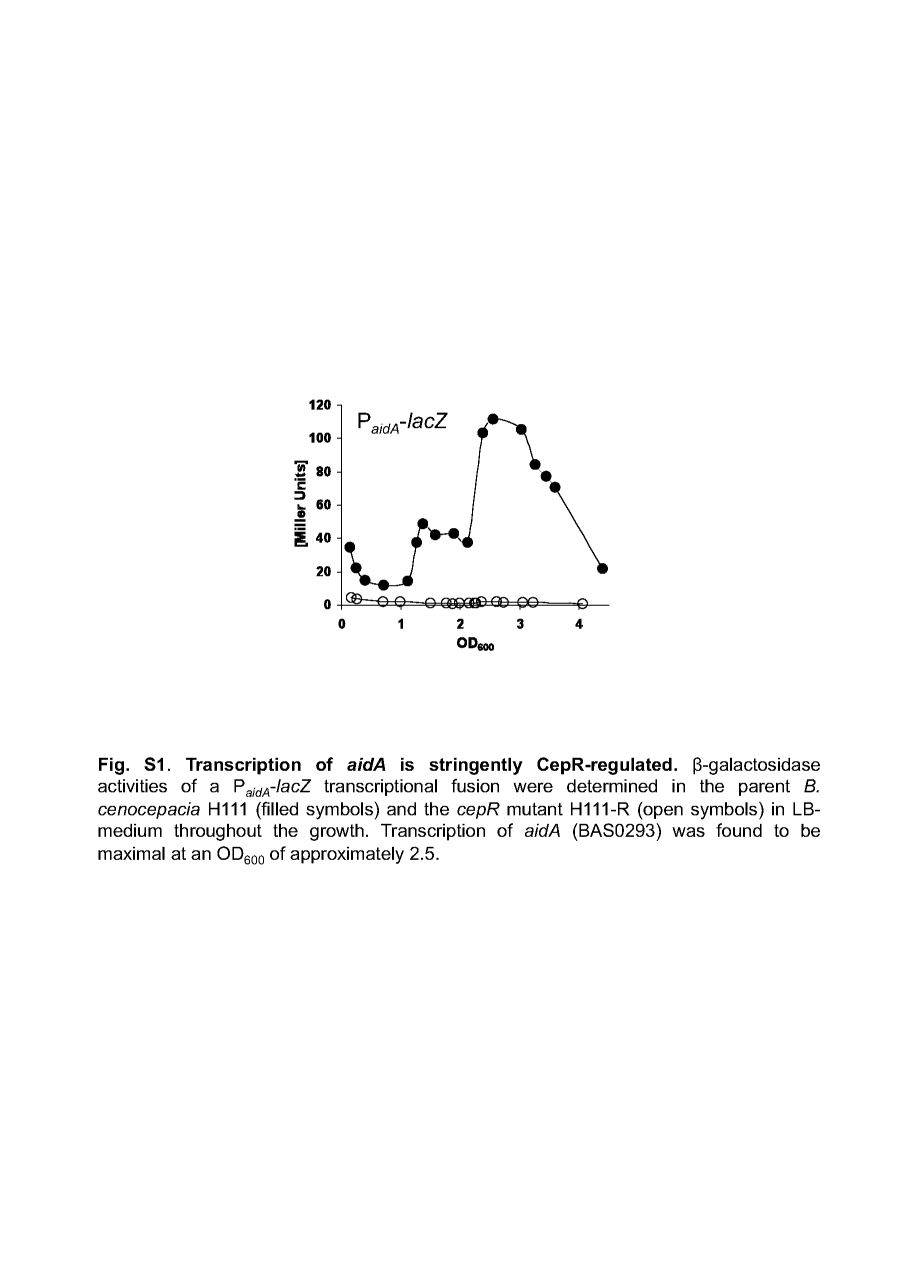

Supplement: Supplementary file 3 [file mbo30001-0225-SD7.png]

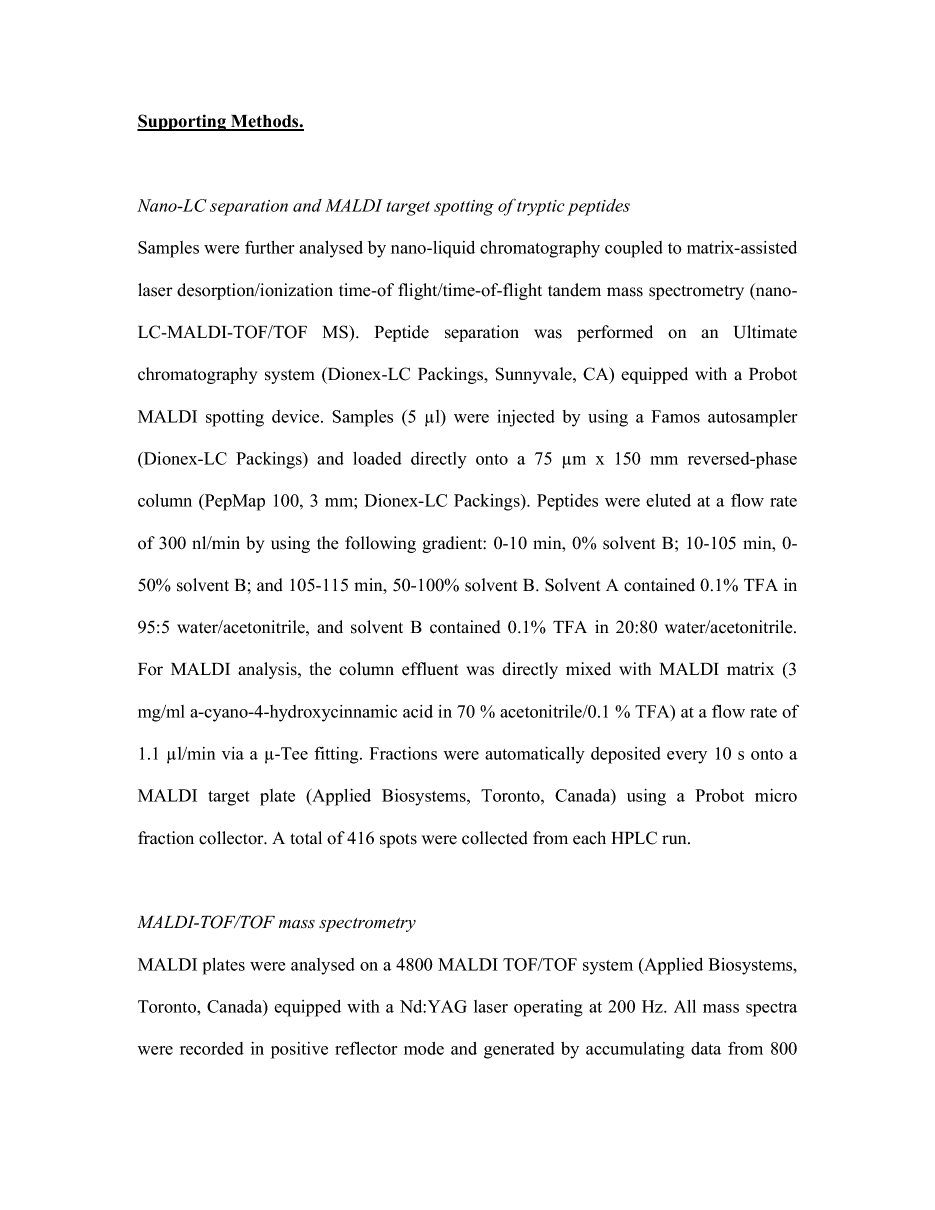

Supplement: Supplementary file 4 [file mbo30001-0225-SD8.png]

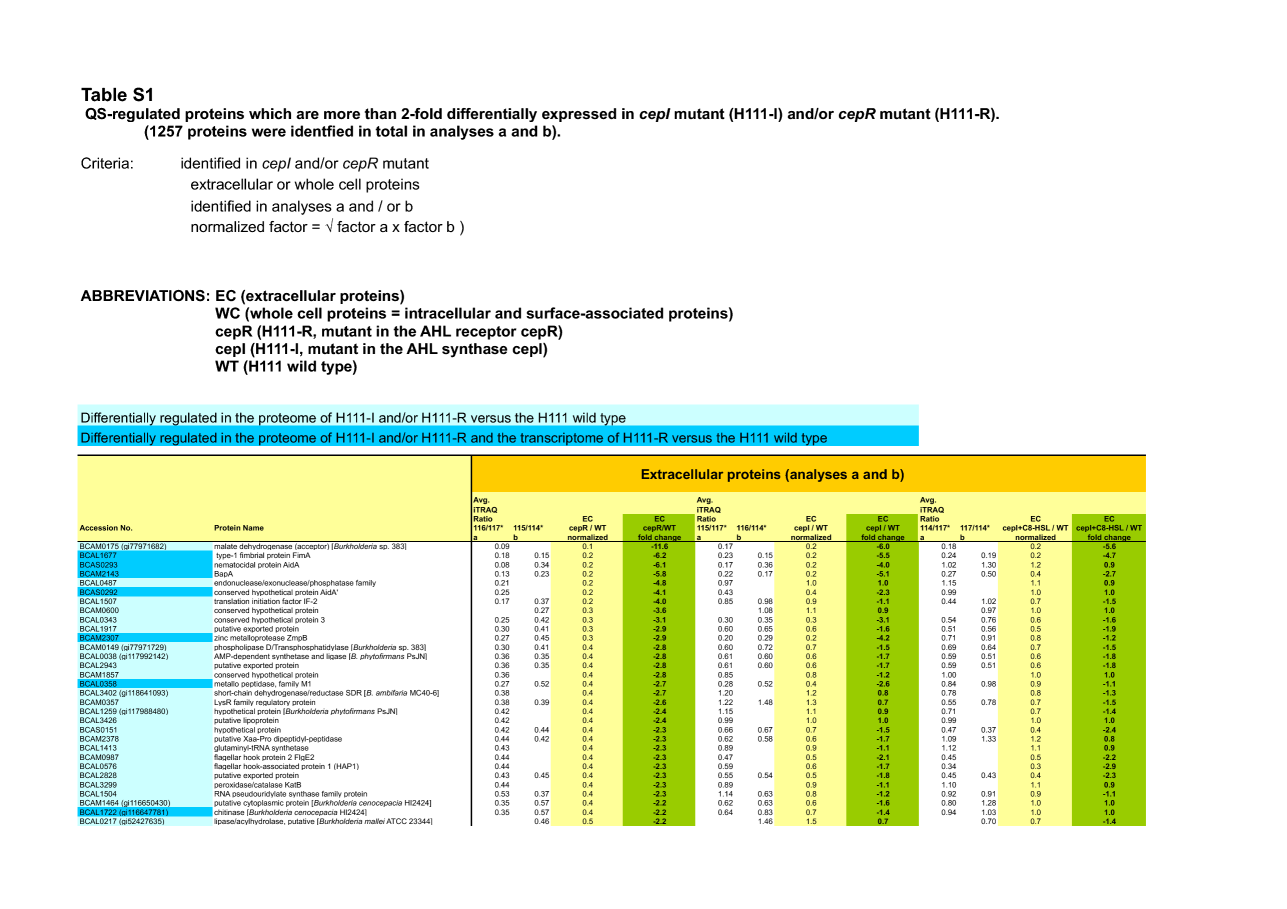

Supplement: Supplementary file 6 [file mbo30001-0225-SD9.png]

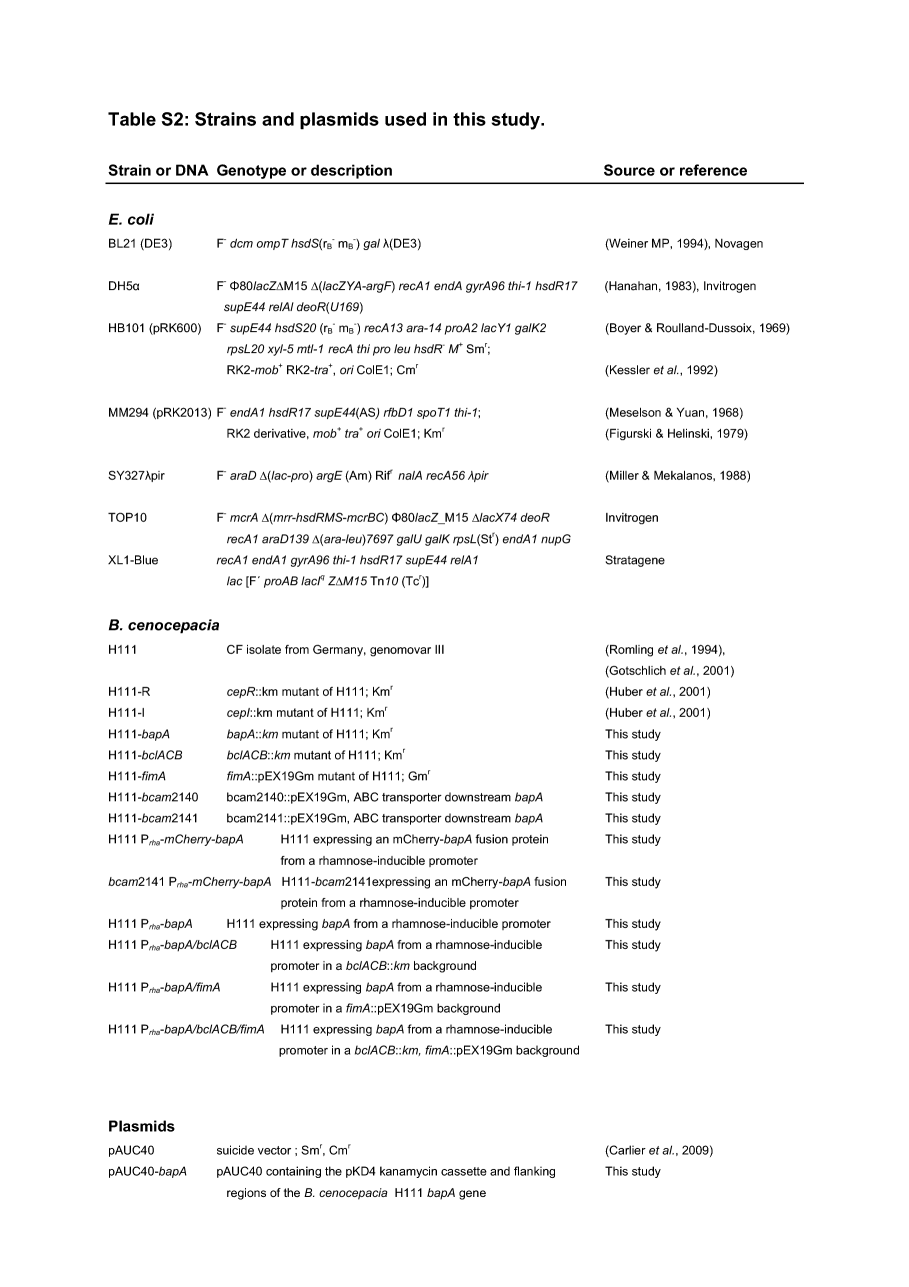

Supplement: Supplementary file 8 [file mbo30001-0225-SD10.png]

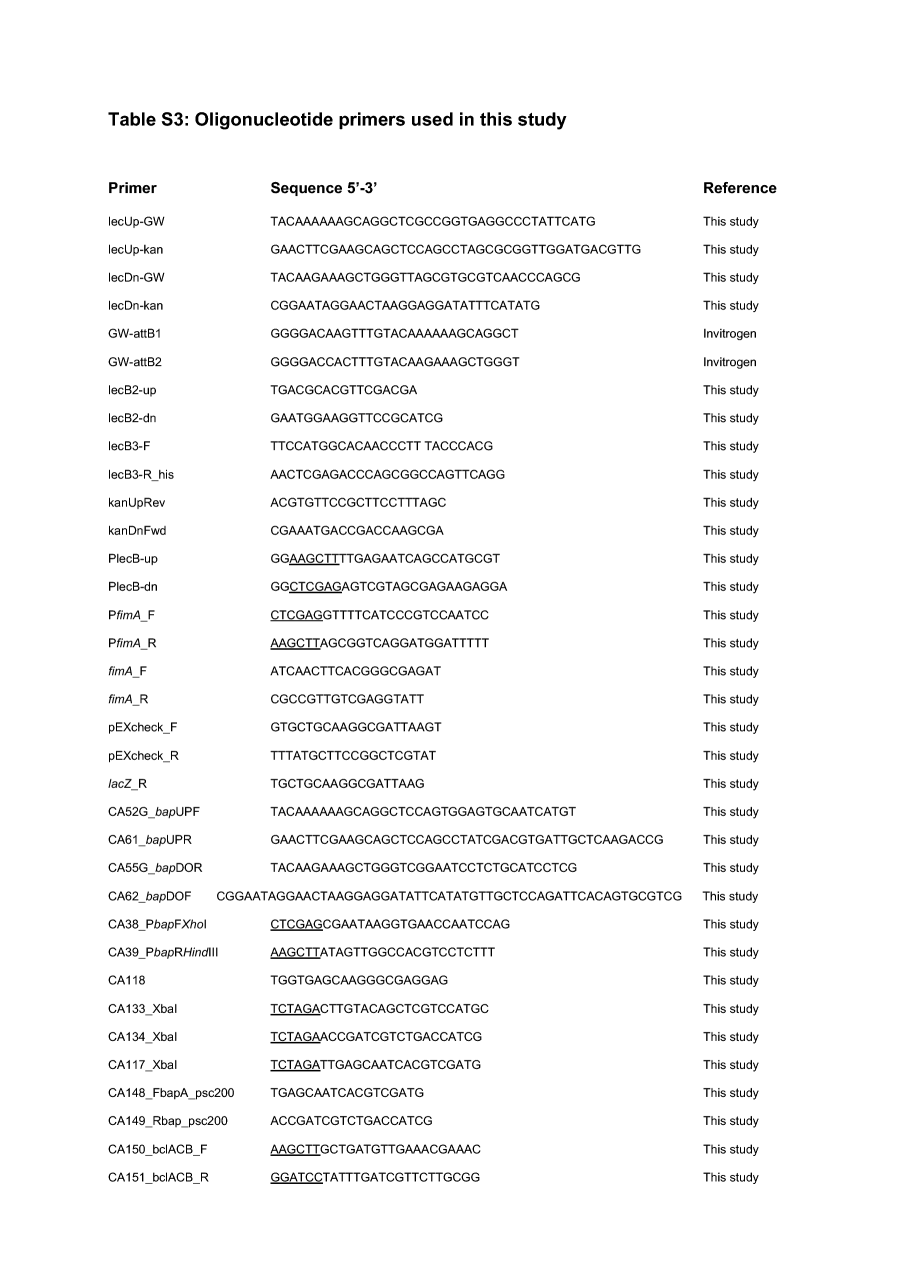

Supplement: Supplementary file 10 [file mbo30001-0225-SD11.png]

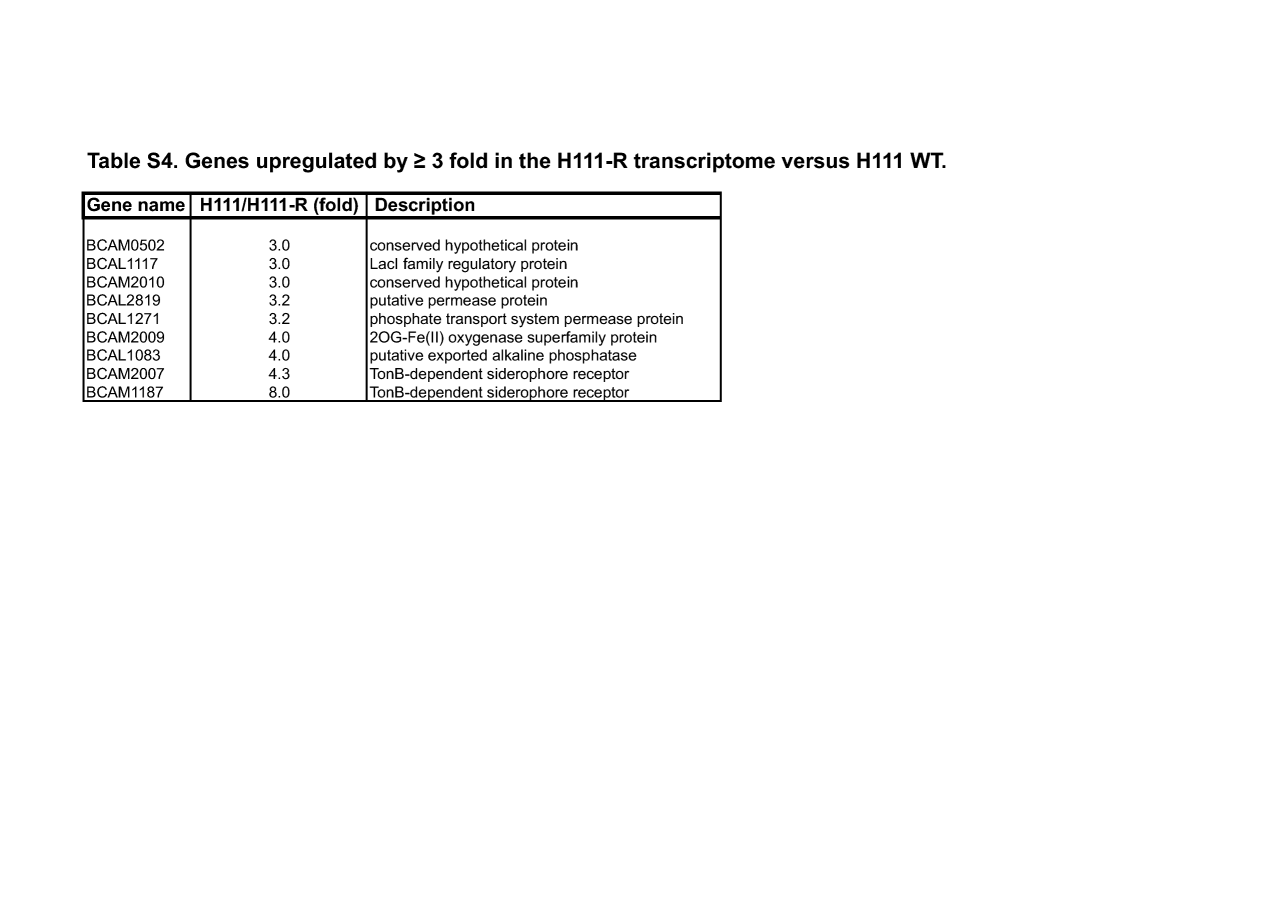

Supplement: Supplementary file 12 [file mbo30001-0225-SD12.png]
