# Supplementary material for: Identification of functions linking quorum sensing with biofilm formation in Burkholderia cenocepacia H111
Source: Microbiologyopen. 2012 Jun;1(2):225–42. doi: 10.1002/mbo3.24 (PMC3426421; doi:10.1002/mbo3.24)
Supplement: Supplementary file 5 [file mbo30001-0225-SD3.pdf]

Table S1

QS-regulated proteins which are more than 2-fold differentially expressed in *cepI* mutant (H111-I) and/or *cepR* mutant (H111-R).
(1257 proteins were identfied in total in analyses a and b).

Criteria: identified in *cepI* and/or *cepR* mutant
extracellular or whole cell proteins
identified in analyses a and / or b
normalized factor = √ factor a x factor b)

ABBREVIATIONS: EC (extracellular proteins)
WC (whole cell proteins = intracellular and surface-associated proteins)
cepR (H111-R, mutant in the AHL receptor cepR)
cepI (H111-I, mutant in the AHL synthase cepI)
WT (H111 wild type)

Differentially regulated in the proteome of H111-I and/or H111-R versus the H111 wild type

Differentially regulated in the proteome of H111-I and/or H111-R and the transcriptome of H111-R versus the H111 wild type

		**Extracellular proteins (analyses a and b)**											
**Accession No.**	**Protein Name**	**Avg. iTRAQ Ratio 116/117* a**	**115/114* b**	**EC cepR / WT normalized**	**EC cepR/WT fold change**	**Avg. iTRAQ Ratio 115/117* a**	**116/114* b**	**EC cepI / WT normalized**	**EC cepI / WT fold change**	**Avg. iTRAQ Ratio 114/117* a**	**117/114* b**	**EC cepI+C8-HSL / WT normalized**	**EC cepI+C8-HSL / WT fold change**
BCAM0175 (gi77971682)	malate dehydrogenase (acceptor) [*Burkholderia* sp. 383]	0.09		0.1	**-11.6**	0.17		0.2	**-6.0**	0.18		0.2	**-5.6**
BCAL1677	type-1 fimbrial protein FimA	0.18	0.15	0.2	**-6.2**	0.23	0.15	0.2	**-5.5**	0.24	0.19	0.2	**-4.7**
BCAS0293	nematocidal protein AidA	0.08	0.34	0.2	**-6.1**	0.17	0.36	0.2	**-4.0**	1.02	1.30	1.2	**0.9**
BCAM2143	BapA	0.13	0.23	0.2	**-5.8**	0.22	0.17	0.2	**-5.1**	0.27	0.50	0.4	**-2.7**
BCAL0487	endonuclease/exonuclease/phosphatase family	0.21		0.2	**-4.8**	0.97		1.0	**1.0**	1.15		1.1	**0.9**
BCAS0292	conserved hypothetical protein AidA'	0.25		0.2	**-4.1**	0.43		0.4	**-2.3**	0.99		1.0	**1.0**
BCAL1507	translation initiation factor IF-2	0.17	0.37	0.2	**-4.0**	0.85	0.98	0.9	**-1.1**	0.44	1.02	0.7	**-1.5**
BCAM0600	conserved hypothetical protein		0.27	0.3	**-3.6**		1.08	1.1	**0.9**		0.97	1.0	**1.0**
BCAL0343	conserved hypothetical protein 3	0.25	0.42	0.3	**-3.1**	0.30	0.35	0.3	**-3.1**	0.54	0.76	0.6	**-1.6**
BCAL1917	putative exported protein	0.30	0.41	0.3	**-2.9**	0.60	0.65	0.6	**-1.6**	0.51	0.56	0.5	**-1.9**
BCAM2307	zinc metalloprotease ZmpB	0.27	0.45	0.3	**-2.9**	0.20	0.29	0.2	**-4.2**	0.71	0.91	0.8	**-1.2**
BCAM0149 (gi77971729)	phospholipase D/Transphosphatidylase [*Burkholderia* sp. 383]	0.30	0.41	0.4	**-2.8**	0.60	0.72	0.7	**-1.5**	0.69	0.64	0.7	**-1.5**
BCAL0038 (gi117992142)	AMP-dependent synthetase and ligase [*B. phytofirmans* PsJN]	0.36	0.35	0.4	**-2.8**	0.61	0.60	0.6	**-1.7**	0.59	0.51	0.6	**-1.8**
BCAL2943	putative exported protein	0.36	0.35	0.4	**-2.8**	0.61	0.60	0.6	**-1.7**	0.59	0.51	0.6	**-1.8**
BCAM1857	conserved hypothetical protein	0.36		0.4	**-2.8**	0.85		0.8	**-1.2**	1.00		1.0	**1.0**
BCAL0358	metallo peptidase, family M1	0.27	0.52	0.4	**-2.7**	0.28	0.52	0.4	**-2.6**	0.84	0.98	0.9	**-1.1**
BCAL3402 (gi118641093)	short-chain dehydrogenase/reductase SDR [*B. ambifaria* MC40-6]	0.38		0.4	**-2.7**	1.20		1.2	**0.8**	0.78		0.8	**-1.3**
BCAM0357	LysR family regulatory protein	0.38	0.39	0.4	**-2.6**	1.22	1.48	1.3	**0.7**	0.55	0.78	0.7	**-1.5**
BCAL1259 (gi117988480)	hypothetical protein [*Burkholderia phytofirmans* PsJN]	0.42		0.4	**-2.4**	1.15		1.1	**0.9**	0.71		0.7	**-1.4**
BCAL3426	putative lipoprotein	0.42		0.4	**-2.4**	0.99		1.0	**1.0**	0.99		1.0	**1.0**
BCAS0151	hypothetical protein	0.42	0.44	0.4	**-2.3**	0.66	0.67	0.7	**-1.5**	0.47	0.37	0.4	**-2.4**
BCAM2378	putative Xaa-Pro dipeptidyl-peptidase	0.44	0.42	0.4	**-2.3**	0.62	0.58	0.6	**-1.7**	1.09	1.33	1.2	**0.8**
BCAL1413	glutaminyl-tRNA synthetase	0.43		0.4	**-2.3**	0.89		0.9	**-1.1**	1.12		1.1	**0.9**
BCAM0987	flagellar hook protein 2 FlgE2	0.44		0.4	**-2.3**	0.47		0.5	**-2.1**	0.45		0.5	**-2.2**
BCAL0576	flagellar hook-associated protein 1 (HAP1)	0.44		0.4	**-2.3**	0.59		0.6	**-1.7**	0.34		0.3	**-2.9**
BCAL2828	putative exported protein	0.43	0.45	0.4	**-2.3**	0.55	0.54	0.5	**-1.8**	0.45	0.43	0.4	**-2.3**
BCAL3299	peroxidase/catalase KatB	0.44		0.4	**-2.3**	0.89		0.9	**-1.1**	1.10		1.1	**0.9**
BCAL1504	RNA pseudouridylate synthase family protein	0.53	0.37	0.4	**-2.3**	1.14	0.63	0.8	**-1.2**	0.92	0.91	0.9	**-1.1**
BCAM1464 (gi116650430)	putative cytoplasmic protein [*Burkholderia cenocepacia* HI2424]	0.35	0.57	0.4	**-2.2**	0.62	0.63	0.6	**-1.6**	0.80	1.28	1.0	**1.0**
BCAL1722 (gi116647781)	chitinase [*Burkholderia cenocepacia* HI2424]	0.35	0.57	0.4	**-2.2**	0.64	0.83	0.7	**-1.4**	0.94	1.03	1.0	**1.0**
BCAL0217 (gi52427635)	lipase/acylhydrolase, putative [*Burkholderia mallei* ATCC 23344]		0.46	0.5	**-2.2**		1.46	1.5	**0.7**		0.70	0.7	**-1.4**

BCAL2888	ribonuclease E 1	0.53	0.40	0.5	**-2.2**	1.27	1.02	1.1	**0.9**	1.11	0.66	0.9	**-1.2**
BCAL1831	putative betaine aldehyde dehydrogenase	0.46		0.5	**-2.2**	0.83		0.8	**-1.2**	1.01		1.0	**1.0**
BCAL2785	putative peptide methionine sulfoxide reductase	0.26	0.87	0.5	**-2.1**	0.51	0.94	0.7	**-1.4**	0.31	0.82	0.5	**-2.0**
BCAM1931	putative porin	0.37	0.62	0.5	**-2.1**	0.93	1.08	1.0	**1.0**	1.04	1.35	1.2	**0.8**
BCAL0980 (gi83653798)	molybdopterin biosynthesis moeA protein [*B. thailandensis* E264]	0.48		0.5	**-2.1**	0.34		0.3	**-2.9**	0.36		0.4	**-2.8**
BCAL3008	putative outer membrane porin protein	0.43	0.54	0.5	**-2.1**	0.82	0.98	0.9	**-1.1**	0.99	1.21	1.1	**0.9**
BCAM0186	lectin BclA	0.45	0.53	0.5	**-2.1**	0.53	0.65	0.6	**-1.7**	0.54	0.63	0.6	**-1.7**
BCAL0742	protein-export protein		0.49	0.5	**-2.0**		1.29	1.3	**0.8**		1.28	1.3	**0.8**
BCAL0567	flagellar hook protein 1 FlgE1	0.43	0.56	0.5	**-2.0**	0.47	0.55	0.5	**-2.0**	0.44	0.48	0.5	**-2.2**
BCAM2308	putative leucyl aminopeptidase precursor	0.49		0.5	**-2.0**	0.53		0.5	**-1.9**	0.66		0.7	**1.5**
BCAL0934	putative periplasmic cytochrome c containing	0.50		0.5	**-2.0**	1.52		1.5	**0.7**	1.15		1.2	**0.9**
BCAL2206	phasin-like protein		0.50	0.5	**-2.0**		0.86	0.9	**-1.2**		1.66	1.7	**0.6**
BCAS0409	zinc metalloprotease ZmpA	0.46	0.55	0.5	**-2.0**	0.45	0.45	0.5	**-2.2**	0.68	0.83	0.8	**-1.3**
BCAL0224	50S ribosomal protein L10	0.51		0.5	**-2.0**	1.17		1.2	**0.9**	1.14		1.1	**0.9**
BCAL3429	putative ribonucleoside reductase	0.44	0.59	0.5	**-2.0**	0.88	1.01	0.9	**-1.1**	1.00	1.10	1.0	**1.0**
BCAL0238	30S ribosomal protein S19		1.95	2.0	**2.0**		0.83	0.8	**-1.2**		1.37	1.4	**0.7**
BCAS0334 (gi134132307)	response regulator receiver protein [*B. vietnamiensis* G4]		1.96	2.0	**2.0**		1.02	1.0	**1.0**		1.31	1.3	**0.8**
BCAL0895	putative peptidyl-prolyl cis-trans isomerase	2.11	1.87	2.0	**2.0**	1.28	1.40	1.3	**1.3**	1.06	1.04	1.0	**1.0**
BCAL2429	putative cytochrome C precursor-related protein	2.00		2.0	**2.0**	1.46		1.5	**1.5**	1.02		1.0	**1.0**
BCAL0247	30S ribosomal protein S14	2.15	1.89	2.0	**2.0**	1.29	1.14	1.2	**1.2**	1.19	1.06	1.1	**1.1**
BCAM2480 (gi52423083)	transcriptional regulator, AsnC family [*B. mallei* ATCC 23344]	1.89	2.26	2.1	**2.1**	1.21	1.28	1.2	**1.2**	0.93	1.07	1.0	**1.0**
BCAL2476a	conserved hypothetical protein (fragment)	2.08	2.06	2.1	**2.1**	1.19	1.33	1.3	**1.3**	0.99	1.06	1.0	**1.0**
BCAL2762	putative adenylate kinase	1.88	2.29	2.1	**2.1**	0.91	1.17	1.0	**1.0**	0.98	0.96	1.0	**1.0**
BCAM2159	putative exported protein	2.17	2.02	2.1	**2.1**	0.82	0.77	0.8	**-1.3**	0.86	0.86	0.9	**-1.2**
BCAM2618	putative periplasmic	2.09	2.13	2.1	**2.1**	0.93	1.17	1.0	**1.0**	1.00	1.01	1.0	**1.0**
BCAL3147	10 kDa chaperonin 1	1.86	2.42	2.1	**2.1**	1.12	1.28	1.2	**1.2**	0.93	1.21	1.1	**1.1**
BCAL0332	putative stringent starvation protein B	2.57	1.78	2.1	**2.1**	0.71	1.10	0.9	**-1.1**	0.63	0.73	0.7	**-1.5**
BCAL1506	N utilization substance protein A	1.97	2.39	2.2	**2.2**	1.28	1.32	1.3	**1.3**	1.13	1.45	1.3	**1.3**
BCAS0010 (gi77965393)	protein of unknown function DUF6, transmembrane [*Burkholderia* sp. 383]	2.49	1.89	2.2	**2.2**	1.39	1.05	1.2	**1.2**	1.15	1.09	1.1	**1.1**
BCAL3054	6,7-dimethyl-8-ribityllumazine synthase	1.63	2.91	2.2	**2.2**	1.03	1.22	1.1	**1.1**	1.07	0.55	0.8	**-1.3**
BCAM0963	putative exported protein	2.11	2.27	2.2	**2.2**	0.94	1.17	1.0	**1.0**	1.04	1.01	1.0	**1.0**
BCAL0935	putative periplasmic cytochrome c protein	2.21		2.2	**2.2**	2.17		2.2	**2.2**	1.49		1.5	**1.5**
BCAL2088	ribosome recycling factor	2.44	2.03	2.2	**2.2**	1.08	1.07	1.1	**1.1**	1.37	0.93	1.1	**1.1**
BCAS0765 (gi77969929) Holden antidi	hydrophobe/amphiphile efflux pump, HAE1 family [*B.* sp. 383]		2.23	2.2	**2.2**		1.99	2.0	**2.0**		1.55	1.5	**1.5**
BCAL3270	putative DnaK chaperone protein	2.36	2.14	2.2	**2.2**	1.02	1.08	1.1	**1.0**	1.23	1.13	1.2	**1.2**
BCAL3378	ferric uptake regulator	3.54	1.45	2.3	**2.3**	1.96	1.04	1.4	**1.4**	1.74	0.70	1.1	**1.1**
BCAL2950	30S ribosomal protein S1	2.53	2.03	2.3	**2.3**	1.18	0.97	1.1	**1.1**	1.18	1.07	1.1	**1.1**
BCAL0799	ribosomal L25p family protein	2.32	2.22	2.3	**2.3**	0.92	1.03	1.0	**1.0**	0.95	0.97	1.0	**1.0**
BCAL2765	putative 30S ribosomal protein S20	2.28		2.3	**2.3**	1.39		1.4	**1.4**	1.99		2.0	**2.0**
BCAM2755	putative exported protein	2.12	2.48	2.3	**2.3**	0.72	0.87	0.8	**-1.3**	0.93	1.08	1.0	**1.0**
BCAL3347	30S ribosomal protein S9	2.24	2.36	2.3	**2.3**	0.99	1.10	1.0	**1.0**	1.10	1.37	1.2	**1.2**
BCAM1443	putative exported protein	2.31		2.3	**2.3**	1.09		1.1	**1.1**	1.06		1.1	**1.1**
BCAL3244 (gi83654978)	possible glycosyltransferase WbpX, putative [*B. thailandensis* E264]	2.32		2.3	**2.3**	0.98		1.0	**1.0**	1.20		1.2	**1.2**
BCAL1044	GntR family regulatory protein	2.32		2.3	**2.3**	1.03		1.0	**1.0**	0.86		0.9	**-1.2**
BCAL1905	50S ribosomal protein L31	2.84	2.06	2.4	**2.4**	1.04	0.89	1.0	**1.0**	0.95	0.88	0.9	**1.1**
BCAM1679 (gi117985553)	protein of unknown function DUF472 [*B. phymatum* STM815]	2.45		2.4	**2.4**	1.25		1.3	**1.3**	1.51		1.5	**1.5**
BCAL2606	two-component regulatory system, response		2.47	2.5	**2.5**		1.90	1.9	**1.9**		1.26	1.3	**1.3**
BCAL1263	transcription elongation factor	2.50		2.5	**2.5**	1.21		1.2	**1.2**	0.93		0.9	**-1.1**
BCAL0245	50S ribosomal protein L24	2.29	2.73	2.5	**2.5**	0.71	0.89	0.8	**-1.3**	0.95	1.23	1.1	**1.1**
BCAL3272	putative heat shock protein	2.51		2.5	**2.5**	1.63		1.6	**1.6**	1.01		1.0	**1.0**
BCAL2958	putative ompA family protein	2.52	2.67	2.6	**2.6**	1.09	1.26	1.2	**1.2**	1.03	1.16	1.1	**1.1**
BCAL1092	ABC transporter extracellular solute-binding	2.60		2.6	**2.6**	1.37		1.4	**1.4**	1.38		1.4	**1.4**
BCAM1062	hypothetical phage protein	2.68		2.7	**2.7**	1.47		1.5	**1.5**	1.28		1.3	**1.3**
xdhA (gi115283038)	transcriptional regulator, LysR family [*B. cepacia* AMMD]	2.68		2.7	**2.7**	1.47		1.5	**1.5**	1.28		1.3	**1.3**
BCAM0833 (gi118660931)	OsmC family protein [*Burkholderia multivorans* ATCC 17616]	2.83	2.62	2.7	**2.7**	1.60	1.47	1.5	**1.5**	1.13	1.06	1.1	**1.1**
BCAL0236	50S ribosomal protein L23	2.74		2.7	**2.7**	0.99		1.0	**1.0**	1.22		1.2	**1.2**
BCAM0915	30S ribosomal protein S21 2	3.00	2.54	2.8	**2.8**	1.68	1.53	1.6	**1.6**	2.33	1.71	2.0	**2.0**
BCAS0773	putative exported protein	2.77		2.8	**2.8**	0.69		0.7	**-1.4**	1.49		1.5	**1.5**
BCAL1943	30S ribosomal protein S18	2.93	2.73	2.8	**2.8**	1.16	1.24	1.2	**1.2**	1.55	1.28	1.4	**1.4**
BCAL0215	phenylacetic acid degradation protein PaaB	3.05	2.64	2.8	**2.8**	1.18	1.29	1.2	**1.2**	1.04	1.14	1.1	**1.1**
BCAL1942	50S ribosomal protein L9	3.28	2.51	2.9	**2.9**	1.45	1.34	1.4	**1.4**	1.87	1.45	1.6	**1.6**
BCAL2022	PspA/IM30 family protein	3.13	2.65	2.9	**2.9**	1.26	0.78	1.0	**1.0**	0.82	0.93	0.9	**-1.1**
BCAM0507	CsbD-like protein	2.99	2.91	3.0	**3.0**	0.60	0.76	0.7	**-1.5**	1.81	1.37	1.6	**1.6**
BCAL1992 (gi77967434)	lipolytic enzyme, G-D-S-L [*Burkholderia* sp. 383]		2.95	3.0	**3.0**		1.22	1.2	**1.2**		0.84	0.8	**1.2**
BCAL3368 (gi117980776)	transcriptional regulator, RpiR family [*B. phymatum* STM815]		2.95	3.0	**3.0**		1.22	1.2	**1.2**		0.84	0.8	**1.2**
BCAL0012	putative adenylate cyclase	3.22	2.80	3.0	**3.0**	1.09	1.50	1.3	**1.3**	0.85	1.12	1.0	**1.0**
BCAL3358	periplasmic glutamate/aspartate-binding protein	2.81	3.40	3.1	**3.1**	1.26	1.33	1.3	**1.3**	1.21	1.50	1.3	**1.3**
BCAM0844	conserved hypothetical protein	2.97	3.32	3.1	**3.1**	1.36	1.41	1.4	**1.4**	1.30	1.14	1.2	**1.2**
BCAL2935	electron transfer flavoprotein beta-subunit	2.47	4.02	3.1	**3.1**	1.08	1.27	1.2	**1.2**	1.50	1.53	1.5	**1.5**
BCAL0043	putative extracellular ligand-binding protein	3.57	2.97	3.3	**3.3**	0.94	1.11	1.0	**1.0**	1.40	1.34	1.4	**1.4**
BCAL2396	conserved hypothetical protein	4.41	2.57	3.4	**3.4**	1.51	1.31	1.4	**1.4**	1.38	1.07	1.2	**1.2**
BCAL0341O	conserved hypothetical protein	2.53	4.50	3.4	**3.4**	1.57	1.68	1.6	**1.6**	1.33	1.99	1.6	**1.6**
BCAL1997	trigger factor	3.31	3.54	3.4	**3.4**	1.00	1.24	1.1	**1.1**	1.23	1.24	1.2	**1.2**
BCAL1985	putative exported isomerase	4.22	2.96	3.5	**3.5**	1.59	1.35	1.5	**1.5**	1.44	1.14	1.3	**1.3**
BCAL2607	putative exported protein		3.55	3.6	**3.6**		0.61	0.6	**-1.6**		0.53	0.5	**-1.9**
BCAL2018 (gi105898280)	transcriptional regulator, GntR family [*B. cenocepacia* AU 1054]	3.56		3.6	**3.6**	0.87		0.9	**-1.2**	1.26		1.3	**1.3**
BCAM0504	CsbD-like protein	3.72	3.46	3.6	**3.6**	1.04	1.10	1.1	**1.1**	0.93	1.04	1.0	**1.0**
BCAM2167	conserved hypothetical protein	3.65		3.6	**3.6**	1.19		1.2	**1.2**	1.05		1.0	**1.0**
BCAL0502	DksA/TraR C4-type zinc finger family protein	2.53	5.26	3.6	**3.6**	1.34	1.73	1.5	**1.5**	1.14	1.53	1.3	**1.3**
BCAL0249	50S ribosomal protein L6	3.83	3.69	3.8	**3.8**	1.15	1.38	1.3	**1.3**	1.17	1.25	1.2	**1.2**
BCAL3427	histone H1-like protein	2.85	4.98	3.8	**3.8**	0.86	1.68	1.2	**1.2**	1.16	1.90	1.5	**1.5**

BCAS0070 (gi118660709)	histidine kinase [*Burkholderia multivorans* ATCC 17616]	4.17		4.2	**4.2**	1.04		1.0	**1.0**	1.14		1.1	**1.1**
BCAS0190	putative H-NS family DNA-binding protein	4.79	3.73	4.2	**4.2**	1.21	1.22	1.2	**1.2**	1.16	0.94	1.0	**1.0**
BCAL0804	putative membrane protein		4.31	4.3	**4.3**		2.04	2.0	**2.0**		1.44	1.4	**1.4**
BCAL0812	sigma-54 modulation protein	5.33	4.14	4.7	**4.7**	1.60	1.42	1.5	**1.5**	1.38	1.07	1.2	**1.2**
BCAL0009	pterin-4-alpha-carbinolamine dehydratase	4.76		4.8	**4.8**	2.03		2.0	**2.0**	1.72		1.7	**1.7**
BCAL0680	conserved hypothetical protein	4.91		4.9	**4.9**	2.12		2.1	**2.1**	1.37		1.4	**1.4**
BCAL1868	conserved hypothetical protein	6.44	3.79	4.9	**4.9**	1.34	1.26	1.3	**1.3**	1.01	0.78	0.9	**-1.1**
BCAL0154	histone-like nucleoid-structuring (H-NS)	5.66		5.7	**5.7**	1.66		1.7	**1.7**	1.36		1.4	**1.4**
BCAL3055	probable N utilization substance protein B		5.78	5.8	**5.8**		1.59	1.6	**1.6**		1.07	1.1	**1.1**
BCAL3530	DNA-binding protein HU-alpha	6.24	6.77	6.5	**6.5**	1.05	1.30	1.2	**1.2**	1.36	1.33	1.3	**1.3**
BCAM1012	putative histone-like protein	6.11	7.26	6.7	**6.7**	1.44	1.80	1.6	**1.6**	1.71	1.73	1.7	**1.7**
BCAM1538	putative dehydrogenase, monooxygenase subunit	7.77	7.29	7.5	**7.5**	2.23	3.18	2.7	**2.7**	3.31	3.38	3.3	**3.3**

		**Intracellular & surface-associated proteins (analyses a & b)**											
**Accession No.**	**Protein Name**	**Avg. iTRAQ Ratio 116/117* a**	**115/114* b**	**WC cepR / WT normalized**	**WC cepR / WT fold change**	**Avg. iTRAQ Ratio 115/117* a**	**116/114* b**	**WC cepI / WT normalized**	**WC cepI / WT fold change**	**Avg. iTRAQ Ratio 114/117* a**	**117/114* b**	**WC cepI+C8-HSL / WT normalized**	**WC cepI+C8-HSL / WT fold change**
BCAS0293	nematocidal protein AidA	0.02	0.06	0.04	**-26.9**	0.04	0.01	0.02	**-47.7**	0.57	0.63	0.6	**-1.7**
BCAS0292	conserved hypothetical protein AidA'		0.26	0.3	**-3.8**		0.18	0.2	**-5.6**		0.74	0.7	**-1.4**
BCAM2308	putative leucyl aminopeptidase precursor		0.27	0.3	**-3.8**		0.29	0.3	**-3.4**		0.82	0.8	**-1.2**
BCAL0831	putative storage protein		0.28	0.3	**-3.5**		0.43	0.4	**-2.3**		0.42	0.4	**-2.4**
BCAM2307	zinc metalloprotease ZmpB	0.25	0.33	0.3	**-3.5**	0.30	0.36	0.3	**-3.1**	0.82	0.80	0.8	**-1.2**
BCAM0026	putative siderophore-interacting protein	0.31		0.3	**-3.2**	0.33		0.3	**-3.1**	0.87		0.9	**-1.1**
BCAM1412 (gi105895814)	conserved hypothetical protein [*Burkholderia cenocepacia* AU 1054]	0.38		0.4	**-2.6**	0.23		0.2	**-4.3**	0.66		0.7	**-1.5**
BCAL3186	conserved hypothetical protein	0.40		0.4	**-2.5**	1.11		1.1	**0.9**	0.85		0.9	**-1.2**
BCAL2226	putative molybdopterin-binding protein		0.43	0.4	**-2.4**		0.55	0.6	**-1.8**		2.22	2.2	**0.5**
BCAL0122	histone-like nucleoid-structuring (H-NS)	0.41	0.48	0.4	**-2.3**	0.42	0.33	0.4	**-2.7**	0.44	0.45	0.4	**-2.3**
BCAM0184	lectin BclB	0.39	0.51	0.4	**-2.3**	0.35	0.55	0.4	**-2.3**	1.00	1.22	1.1	**0.9**
BCAL0347	protease associated ATPase ClpB	0.45		0.5	**-2.2**	0.39		0.4	**-2.6**	0.80		0.8	**-1.3**
BCAL2941	putative exported transglycosylase		0.46	0.5	**-2.2**		0.56	0.6	**-1.8**		0.41	0.4	**-2.4**
BCAM2006	putative aspartate carbomyltransferase		0.47	0.5	**-2.1**		1.63	1.6	**0.6**		0.96	1.0	**1.0**
BCAL2354	2-isopropylmalate synthase	0.48	0.47	0.5	**-2.1**	0.70	0.49	0.6	**-1.7**	1.07	0.67	0.8	**-1.2**
BCAL0360	conserved hypothetical protein	0.72	0.32	0.5	**-2.1**	0.95	0.87	0.9	**-1.1**	0.86	0.95	0.9	**-1.1**
BCAL0704	D-alanyl-D-alanine carboxypeptidase		0.49	0.5	**-2.0**		0.69	0.7	**-1.4**		0.85	0.8	**-1.2**
BCAL2864	hypothetical protein		0.51	0.5	**-2.0**		0.53	0.5	**-1.9**		0.69	0.7	**-1.5**
BCAL2123	conserved hypothetical protein		1.84	1.8	**1.8**		1.62	1.6	**1.6**		1.49	1.5	**1.5**
BCAL0617	conserved hypothetical protein	1.86		1.9	**1.9**	0.95		0.9	**-1.1**	1.39		1.4	**1.4**
BCAM1491 (gi116650457)	conserved hypothetical protein [*Burkholderia cenocepacia* HI2424]		1.86	1.9	**1.9**		1.77	1.8	**1.8**		0.61	0.6	**-1.7**
BCAL0562	negative regulator of flagellin synthesis		1.89	1.9	**1.9**		1.67	1.7	**1.7**		1.44	1.4	**1.4**
BCAL0349	putative outer membrane protein		1.93	1.9	**1.9**		1.48	1.5	**1.5**		1.58	1.6	**1.6**
BCAL2180	putative 2-dehydro-3-deoxyphosphooctonate	1.89	1.98	1.9	**1.9**	1.18	1.39	1.3	**1.3**	0.81	1.01	0.9	**-1.1**
BCAL0247	30S ribosomal protein S14		2.00	2.0	**2.0**		0.79	0.8	**-1.3**		1.13	1.1	**1.1**
BCAL2796 (gi118659676)	aldehyde dehydrogenase [*B. multivorans* ATCC 17616]	2.02		2.0	**2.0**	3.54		3.5	**3.5**	0.62		0.6	**-1.6**
BCAL0690	conserved hypothetical protein	2.35		2.3	**2.3**	2.67		2.7	**2.7**	1.83		1.8	**1.8**
BCAL3051	riboflavin synthase alpha chain	2.87		2.9	**2.9**	1.68		1.7	**1.7**	2.45		2.5	**2.5**
BCAM0589	conserved hypothetical protein		3.91	3.9	**3.9**		3.94	3.9	**3.9**		2.91	2.9	**2.9**
BCAL3377	putative outer membrane protein		4.04	4.0	**4.0**		3.39	3.4	**3.4**		1.71	1.7	**1.7**
BCAL1869	putative exported protein		4.54	4.5	**4.5**		1.78	1.8	**1.8**		2.86	2.9	**2.9**
