# Supplementary material for: Identification of functions linking quorum sensing with biofilm formation in Burkholderia cenocepacia H111
Source: Microbiologyopen. 2012 Jun;1(2):225–42. doi: 10.1002/mbo3.24 (PMC3426421; doi:10.1002/mbo3.24)
Supplement: Supplementary file 7 [file mbo30001-0225-SD4.pdf]

Table S2: Strains and plasmids used in this study.

Strain or DNA	Genotype or description	Source or reference
E. coli		
BL21 (DE3)	F$^-$ *dcm ompT hsdS*(r_B^- m_B^-) *gal* λ(DE3)	(Weiner MP, 1994), Novagen
DH5α	F$^-$ Φ80*lacZ*ΔM15 Δ(*lacZYA-argF*) *recA1 endA gyrA96 thi-1 hsdR17 supE44 relAl deoR*(*U169*)	(Hanahan, 1983), Invitrogen
HB101 (pRK600)	F$^-$ *supE44 hsdS20* (r_B^- m_B^-) *recA13 ara-14 proA2 lacY1 galK2 rpsL20 xyl-5 mtl-1 recA thi pro leu hsdR$^-$ M$^+$* Sm^r; RK2-*mob*$^+$ RK2-*tra*$^+$, *ori* ColE1; Cm^r	(Boyer & Roulland-Dussoix, 1969) (Kessler *et al.*, 1992)
MM294 (pRK2013)	F$^-$ *endA1 hsdR17 supE44*(AS*) rfbD1 spoT1 thi-1*; RK2 derivative, *mob*$^+$ *tra*$^+$ *ori* ColE1; Km^r	(Meselson & Yuan, 1968) (Figurski & Helinski, 1979)
SY327λpir	F$^-$ *araD* Δ(*lac-pro*) *argE* (Am) Rif^r *nalA recA56 λpir*	(Miller & Mekalanos, 1988)
TOP10	F$^-$ *mcrA* Δ(*mrr-hsdRMS-mcrBC*) Φ80*lacZ*_M15 Δ*lacX74 deoR recA1 araD139* Δ(*ara-leu*)*7697 galU galK rpsL*(St^r) *endA1 nupG*	Invitrogen
XL1-Blue	*recA1 endA1 gyrA96 thi-1 hsdR17 supE44 relA1 lac* [F′ *proAB lacI^q* Z*ΔM15* Tn*10* (Tc^r)]	Stratagene
B. cenocepacia		
H111	CF isolate from Germany, genomovar III	(Romling *et al.*, 1994), (Gotschlich *et al.*, 2001)
H111-R	*cepR*::km mutant of H111; Km^r	(Huber *et al.*, 2001)
H111-I	*cepI*::km mutant of H111; Km^r	(Huber *et al.*, 2001)
H111-*bapA*	*bapA*::*km* mutant of H111; Km^r	This study
H111-*bclACB*	*bclACB*::*km* mutant of H111; Km^r	This study
H111-*fimA*	*fimA*::pEX19Gm mutant of H111; Gm^r	This study
H111-*bcam*2140	bcam2140::pEX19Gm, ABC transporter downstream *bapA*	This study
H111-*bcam*2141	bcam2141::pEX19Gm, ABC transporter downstream *bapA*	This study
H111 P_{rha}-*mCherry-bapA*	H111 expressing an mCherry-*bapA* fusion protein from a rhamnose-inducible promoter	This study
*bcam*2141 P_{rha}-*mCherry-bapA*	H111-*bcam*2141expressing an mCherry-*bapA* fusion protein from a rhamnose-inducible promoter	This study
H111 P_{rha}-*bapA*	H111 expressing *bapA* from a rhamnose-inducible promoter	This study
H111 P_{rha}-*bapA/bclACB*	H111 expressing *bapA* from a rhamnose-inducible promoter in a *bclACB*::*km* background	This study
H111 P_{rha}-*bapA/fimA*	H111 expressing *bapA* from a rhamnose-inducible promoter in a *fimA*::pEX19Gm background	This study
H111 P_{rha}-*bapA/bclACB/fimA*	H111 expressing *bapA* from a rhamnose-inducible promoter in a *bclACB*::*km, fimA*::pEX19Gm background	This study
**Plasmids**		
pAUC40	suicide vector ; Sm^r, Cm^r	(Carlier *et al.*, 2009)
pAUC40-*bapA*	pAUC40 containing the pKD4 kanamycin cassette and flanking regions of the *B. cenocepacia* H111 *bapA* gene	This study

pAUC40-*bcl*	pAUC40 containing the pKD4 kanamycin cassette and flanking regions of the *B. cenocepacia* H111 *bcl* operon (*bclA*-*C*)	This study
pBAH7	pBBR1MCS-2 containing PA1/04/03-*gfp* mut3-To-T1; Km^r	B. Huber (unpublished)
pBAH8	pBBR1MCS-5 containing PA1/04/03-*gfp* mut3-To-T1; Gm^r	(Huber *et al.*, 2002)
pBAH27	pBBR1MCS-5 containing the *cepR* gene of *B. cepacia* H111	(Huber *et al.*, 2001)
pBBR1MCS	broad host-range cloning vector; Cm^r	(Kovach *et al.*, 1994)
pBBR1MCS-2	broad host-range cloning vector; Km^r	(Kovach *et al.*, 1995)
pBBR1MCS-5	broad host-range cloning vector; Gm^r	(Kovach et al., 1995)
pBBR-cepR	pBBR1MCS containing the *cepR* gene of *B. cenocepacia* H111; Cm^r	S. Schmidt (unpublished)
pCR2.1	cloning vector for PCR products; Amp^r, Km^r	Invitrogen
pDONR221	cloning vector; Km^r	Invitrogen
pEX18Gm	*oriT*$^+$ *sacB*$^+$*;* pUC18 MCS, gene replacement vector; Gm^r	(Hoang *et al.*, 1998)
pEX19Gm	*oriT*$^+$*, sacB*$^+$, pUC19 MCS, gene replacement vector; Gm^r	(Hoang et al., 1998)
pET28a	expression vector, T7 promoter; Km^r	Novagen
pET-HisBclB	pET28a derivate carrying the *bclC* gene of *B. cenocepacia* H111; Km^r	This study
pKD4	kanamycin cassette template; Amp^r, Km^r	(Datsenko & Wanner, 2000)
pRN3	promoter probe vector, pSU11 derivative; Tp^r	(Malott et al., 2009)
pRK2013	RK2 derivative, *mob*$^+$ *tra*$^+$ *ori* ColE1; Km^r	(Figurski & Helinski, 1979)
pSU11	promoter probe vector; Gm^r	L. Eberl
P_{fimA}-*lacZ*	pSU11 containing the putative *fimA* promoter region	This study
P_{bapA}-*lacZ*	pSU11 containing the putative *bapA* promoter region	This study
P_{bclA}-*lacZ*	pSU11 containing the putative *bclA* promoter region	This study
pNS1	pEX19Gm containing a 300bp internal fragment of the *B. cenocepacia* H111 *fimA* gene	This study
pSC200	for delivery of rhamnose-inducible *PrhaB* promoter into the chromosome to drive the expression of a targeted gene.	(Ortega *et al.*, 2007)

Antibiotic-resistance of strains or plasmids: ampicillin (Amp^r), chloramphenicol (Cm^r), gentamicin (Gm^r), kanamycin (Km^r), streptomycin (Sm^r) and trimethoprim (Tp^r).

References

Boyer, H. W. & D. Roulland-Dussoix, (1969) A complementation analysis of the restriction and modification of DNA in Escherichia coli. *J Mol Biol* **41**: 459-472.

Carlier, A., L. Burbank & S. B. von Bodman, (2009) Identification and characterization of three novel EsaI/EsaR quorum-sensing controlled stewartan exopolysaccharide biosynthetic genes in Pantoea stewartii ssp. stewartii. *Mol Microbiol* **74**: 903-913.

Datsenko, K. A. & B. L. Wanner, (2000) One-step inactivation of chromosomal genes in Escherichia coli K-12 using PCR products. *Proc Natl Acad Sci U S A* **97**: 6640-6645.

Figurski, D. H. & D. R. Helinski, (1979) Replication of an origin-containing derivative of plasmid RK2 dependent on a plasmid function provided in trans. *Proc Natl Acad Sci U S A* **76**: 1648-1652.

Gotschlich, A., B. Huber, O. Geisenberger, A. Togl, A. Steidle, K. Riedel, P. Hill, B. Tummler, P. Vandamme, B. Middleton, M. Camara, P. Williams, A. Hardman & L. Eberl, (2001) Synthesis of multiple N-acylhomoserine lactones is wide-spread among the members of the Burkholderia cepacia complex. *Syst Appl Microbiol* **24**: 1-14.

Hanahan, D., (1983) Studies on transformation of Escherichia coli with plasmids. *J Mol Biol* **166**: 557-580.

Hoang, T. T., R. R. Karkhoff-Schweizer, A. J. Kutchma & H. P. Schweizer, (1998) A broad-host-range Flp-FRT recombination system for site-specific excision of chromosomally-located DNA sequences: application for isolation of unmarked Pseudomonas aeruginosa mutants. *Gene* **212**: 77-86.

Huber, B., K. Riedel, M. Hentzer, A. Heydorn, A. Gotschlich, M. Givskov, S. Molin & L. Eberl, (2001) The cep quorum-sensing system of Burkholderia cepacia H111 controls biofilm formation and swarming motility. *Microbiology* **147**: 2517-2528.

Huber, B., K. Riedel, M. Kothe, M. Givskov, S. Molin & L. Eberl, (2002) Genetic analysis of functions involved in the late stages of biofilm development in Burkholderia cepacia H111. *Mol Microbiol* **46**: 411-426.

Kessler, B., V. de Lorenzo & K. N. Timmis, (1992) A general system to integrate lacZ fusions into the chromosomes of gram-negative eubacteria: regulation of the Pm promoter of the TOL plasmid studied with all controlling elements in monocopy. *Mol Gen Genet* **233**: 293-301.

Kovach, M. E., P. H. Elzer, D. S. Hill, G. T. Robertson, M. A. Farris, R. M. Roop, 2nd & K. M. Peterson, (1995) Four new derivatives of the broad-host-range cloning vector pBBR1MCS, carrying different antibiotic-resistance cassettes. *Gene* **166**: 175-176.

Kovach, M. E., R. W. Phillips, P. H. Elzer, R. M. Roop, 2nd & K. M. Peterson, (1994) pBBR1MCS: a broad-host-range cloning vector. *Biotechniques* **16**: 800-802.

Malott, R. J., E. P. O'Grady, J. Toller, S. Inhulsen, L. Eberl & P. A. Sokol, (2009) A Burkholderia cenocepacia orphan LuxR homolog is involved in quorum-sensing regulation. *J Bacteriol* **191**: 2447-2460.

Meselson, M. & R. Yuan, (1968) DNA restriction enzyme from E. coli. *Nature* **217**: 1110-1114.

Miller, V. L. & J. J. Mekalanos, (1988) A novel suicide vector and its use in construction of insertion mutations: osmoregulation of outer membrane proteins and virulence determinants in Vibrio cholerae requires toxR. *J Bacteriol* **170**: 2575-2583.

Ortega, X. P., S. T. Cardona, A. R. Brown, S. A. Loutet, R. S. Flannagan, D. J. Campopiano, J. R. Govan & M. A. Valvano, (2007) A putative gene cluster for aminoarabinose biosynthesis is essential for Burkholderia cenocepacia viability. *J Bacteriol* **189**: 3639-3644.

Romling, U., B. Fiedler, J. Bosshammer, D. Grothues, J. Greipel, H. von der Hardt & B. Tummler, (1994) Epidemiology of chronic Pseudomonas aeruginosa infections in cystic fibrosis. *J Infect Dis* **170**: 1616-1621.

Weiner MP, A. C., Jerpseth B, Wells S, Johnson-Browne B, Vaillancourt P (1994) Studier pET system vectors and hosts. *Strateg Mol Biol* **7**: 41–43
