# Supplementary material for: Identification of functions linking quorum sensing with biofilm formation in Burkholderia cenocepacia H111
Source: Microbiologyopen. 2012 Jun;1(2):225–42. doi: 10.1002/mbo3.24 (PMC3426421; doi:10.1002/mbo3.24)
Supplement: Supplementary file 9 [file mbo30001-0225-SD5.pdf]

Table S3: Oligonucleotide primers used in this study

Primer	Sequence 5'-3'	Reference
lecUp-GW	TACAAAAAAGCAGGCTCGCCGGTGAGGCCCTATTCATG	This study
lecUp-kan	GAACTTCGAAGCAGCTCCAGCCTAGCGCGGTTGGATGACGTTG	This study
lecDn-GW	TACAAGAAAGCTGGGTTAGCGTGCGTCAACCCAGCG	This study
lecDn-kan	CGGAATAGGAACTAAGGAGGATATTTCATATG	This study
GW-attB1	GGGGACAAGTTTGTACAAAAAAGCAGGCT	Invitrogen
GW-attB2	GGGGACCACTTTGTACAAGAAAGCTGGGT	Invitrogen
lecB2-up	TGACGCACGTTCGACGA	This study
lecB2-dn	GAATGGAAGGTTCCGCATCG	This study
lecB3-F	TTCCATGGCACAACCCTT TACCCACG	This study
lecB3-R_his	AACTCGAGACCCAGCGGCCAGTTCAGG	This study
kanUpRev	ACGTGTTCCGCTTCCTTTAGC	This study
kanDnFwd	CGAAATGACCGACCAAGCGA	This study
PlecB-up	GGAAGCTTTTGAGAATCAGCCATGCGT	This study
PlecB-dn	GGCTCGAGAGTCGTAGCGAGAAGAGGA	This study
P*fimA*_F	CTCGAGGTTTTCATCCCGTCCAATCC	This study
P*fimA*_R	AAGCTTAGCGGTCAGGATGGATTTTT	This study
*fimA*_F	ATCAACTTCACGGGCGAGAT	This study
*fimA*_R	CGCCGTTGTCGAGGTATT	This study
pEXcheck_F	GTGCTGCAAGGCGATTAAGT	This study
pEXcheck_R	TTTATGCTTCCGGCTCGTAT	This study
*lacZ*_R	TGCTGCAAGGCGATTAAG	This study
CA52G_*bap*UPF	TACAAAAAAGCAGGCTCCAGTGGAGTGCAATCATGT	This study
CA61_*bap*UPR	GAACTTCGAAGCAGCTCCAGCCTATCGACGTGATTGCTCAAGACCG	This study
CA55G_*bap*DOR	TACAAGAAAGCTGGGTCGGAATCCTCTGCATCCTCG	This study
CA62_*bap*DOF	CGGAATAGGAACTAAGGAGGATATTCATATGTTGCTCCAGATTCACAGTGCGTCG	This study
CA38_P*bap*F*Xho*I	CTCGAGCGAATAAGGTGAACCAATCCAG	This study
CA39_P*bap*R*Hind*III	AAGCTTATAGTTGGCCACGTCCTCTTT	This study
CA118	TGGTGAGCAAGGGCGAGGAG	This study
CA133_XbaI	TCTAGACTTGTACAGCTCGTCCATGC	This study
CA134_XbaI	TCTAGAACCGATCGTCTGACCATCG	This study
CA117_XbaI	TCTAGATTGAGCAATCACGTCGATG	This study
CA148_FbapA_psc200	TGAGCAATCACGTCGATG	This study
CA149_Rbap_psc200	ACCGATCGTCTGACCATCG	This study
CA150_bclACB_F	AAGCTTGCTGATGTTGAAACGAAAC	This study
CA151_bclACB_R	GGATCCTATTTGATCGTTCTTGCGG	This study

CA154_bclAC_F	CGATCAACGGCAAGAAGTC	This study
CA155_bclAC_R	GAAATAGATGCCGGGGAAG	This study
CA156_bclCB_F	CACGAAAGTGCTCGATTC	This study
CA157_bclCB_R	TTTCACCTTGTTCTGCCA	This study
CA168_2143_42F	ATCTGTCGAAGCTGCTGGTC	This study
CA169_2143_42R	TCGAACGAATCGGTCAGTC	This study
CA170_2142_41F	CGACCTGTACTGGCAGGAAT	This study
CA171_2142_41R	GATACGATCACGCCGACAT	This study
CA172_2141_40F	GAACGTGTTGCTGCTCGAC	This study
CA173_2141_40R	GATGACCTGCCCTTTCGAT	This study
CA174_1677_78F	GGGCGAATACCTCGACAAC	This study
CA175_1677_78R	CTGCACGTCGAGCATGTACT	This study
CA176_1678_79F	CAACCTGTACCTGACGCTGA	This study
CA177_1678_79R	GCTCTTGTTGGTGATCGTGA	This study
CA178_1679_80F	GAGCCGACGATGGTGAGT	This study
CA179_1679_80R	CCCGCCGTAGTCCTTGTAG	This study
CA182_1680_81F	AGCCGATCGACTTCAACAAG	This study
CA183_1680_81R	ATCACGGTCAGCGAATAACC	This study
CA180_bclB_F_HindIII	AAGCTTCGTTCGGGCAGTCATTT	This study
CA181_bclB_R_BamHI	GGATCCTCGGCGGATGGGTATTTG	This study
AS02_bclAC-R_BamHI	GGATCCGTACAGCAGTGGGACTGCAA	This study
AS03_bclA-R_BamHI	GGATCCCTGACAATCGAAGCCGAAAG	This study

Restriction endonuclease sites are underlined on sequence.
