# Supplementary material for: Identification of functions linking quorum sensing with biofilm formation in Burkholderia cenocepacia H111
Source: Microbiologyopen. 2012 Jun;1(2):225–42. doi: 10.1002/mbo3.24 (PMC3426421; doi:10.1002/mbo3.24)
Supplement: Supplementary file 11 [file mbo30001-0225-SD6.pdf]

Table S4. Genes upregulated by ≥ 3 fold in the H111-R transcriptome versus H111 WT.

Gene name	H111/H111-R (fold)	Description
BCAM0502	3.0	conserved hypothetical protein
BCAL1117	3.0	LacI family regulatory protein
BCAM2010	3.0	conserved hypothetical protein
BCAL2819	3.2	putative permease protein
BCAL1271	3.2	phosphate transport system permease protein
BCAM2009	4.0	2OG-Fe(II) oxygenase superfamily protein
BCAL1083	4.0	putative exported alkaline phosphatase
BCAM2007	4.3	TonB-dependent siderophore receptor
BCAM1187	8.0	TonB-dependent siderophore receptor
